# Nanoemulsions loaded with ß-caryophyllene and resveratrol for herpes infections

**DOI:** 10.1590/0074-02760250333

**Published:** 2026-10-12

**Authors:** Isabella Dai Prá, Douglas Santos Porto, Bianca Costa Bernardo Port, Luíse Gauer Schulte, Débora Fretes Argenta, Thiago Caon, Izabella Thaís Silva

**Affiliations:** 1 Universidade Federal de Santa Catarina, Departamento de Ciências Farmacêuticas, Florianópolis, SC, Brasil; 2 Universidade Federal de Santa Catarina, Departamento de Microbiologia, Imunologia e Parasitologia, Florianopolis, SC, Brasil

**Keywords:** antiviral activity, herpes treatment, drug delivery systems, nanoencapsulation

## Abstract

**BACKGROUND:**

Herpes Simplex Virus (HSV) infection represents a global health concern with a significant impact on affected individuals’ quality of life. Conventional treatment relies on nucleoside analogues such as acyclovir, which, although effective, have limitations including viral resistance and low bioavailability. In this context, natural products such as resveratrol (RSV) and β-caryophyllene (BCP) emerge as promising alternatives due to their antiviral and immunomodulatory properties. However, these compounds present challenges related to stability and solubility, necessitating the use of controlled drug delivery systems such as nanoemulsions.

**OBJECTIVES:**

This study aimed to develop and characterise nanoemulsions containing RSV and BCP, either individually or in combination, for the treatment of HSV type 1 (HSV-1) infections.

**METHODS:**

The nanoemulsions were prepared using a low-energy spontaneous emulsification technique and characterised for particle size, polydispersity index (PDI), and zeta potential. The antiviral activity, cytotoxicity, and wound-healing potential of the nanoemulsions were evaluated *in vitro* using VERO and human gingival fibroblast (HGF) cells.

**FINDINGS:**

Results showed that RSV exhibited significant antiviral activity against HSV-1, while BCP demonstrated moderate activity. Nanoencapsulation improved the biocompatibility of RSV but increased the cytotoxicity of BCP. The combination of RSV and BCP in nanoemulsions showed enhanced antiviral activity, with inhibition percentages above 70%. However, the nanoemulsions did not significantly promote wound healing in HGF cells. Additionally, the nanoemulsions did not protect against Ultraviolet Crosslinker (UVC)-induced cell damage.

**MAIN CONCLUSIONS:**

Nanoemulsions containing RSV and BCP demonstrated enhanced antiviral activity against HSV-1, particularly when combined, suggesting a synergistic effect between both compounds. Nanoencapsulation improved the biocompatibility of RSV but increased the cytotoxicity of BCP. Although no significant effects were observed in wound healing or UV protection assays, the results support nanoemulsions as a promising platform for improving the antiviral performance of these compounds for topical HSV-1 treatment.

The Herpes Simplex virus type 1 (HSV-1) is highly transmissible human pathogen, affecting approximately 3.7 billion individuals under 50 years worldwide. ^1^ HSV-1 initially infects epithelial cells and establishes latency in the neurons of the trigeminal ganglion. ^2^ Factors such as stress, sunlight exposure, and immunosuppression can trigger viral reactivation, leading to the appearance of characteristic mucocutaneous lesions in the orofacial region. ^3^ Exposure to UV radiation, in addition to being one of the main triggers for HSV-1 reactivation, can exacerbate skin lesions, resulting in slower healing and a higher likelihood of complications, such as secondary infections. The mucocutaneous lesions, typical of HSV-1 infection, primarily affect the orofacial region but can extend to other areas of the skin and mucous membranes. In more severe cases, HSV-1 can lead to complications such as encephalitis. ^4^ ^,^ ^5^ ^,^ ^6^

HSV infections are treated primarily with nucleoside analogues such as acyclovir, valacyclovir, and famciclovir, which suppress viral replication but do not eliminate latent infection. Long-term suppressive therapy, recommended for patients with frequent recurrences (≥ 6 episodes/year) and for immunocompromised individuals, is associated with the emergence of drug-resistant strains, which have been reported in up to 5% of immunocompetent patients and in up to 30% of immunocompromised individuals, such as those with human immunodeficiency virus/acquired immunodeficiency syndrome (HIV/AIDS) or transplant recipients.^7^ ^,^ ^8^ This scenario reinforces the urgent need for new antiviral strategies with alternative mechanisms of action. The need for new antiviral therapies against HSV has been explicitly recognised by the World Health Organization (WHO).^1^ In this context, a diversity of plant-based compounds has been investigated as potential anti-herpetic agents, as they frequently exhibit favourable tolerability profiles and may offer complementary or alternative mechanisms of action to conventional antivirals.^9^ ^,^ ^10^ ^,^ ^11^ ^,^ ^11^

Resveratrol (RSV), a polyphenol found in grapes, berries and peanuts, has already been shown to be active against HSV-1. It is thought to act on the ICP-4 viral protein, resulting in a reduction in replication and viral load. Furthermore, the reactivation of latent virus in neuronal cells is prevented as the cell cycle is delayed.^12^ ^,^ ^13^

β-caryophyllene (BCP), a bicyclic sesquiterpene found in *Eugenia caryophyllata*, *Piper nigrum*, *Zingiber nimmonii*, *Copaifera reticulata* and *Cannabis* spp., has shown promising potential in the treatment of viral infections.^14^ ^,^ ^15^ ^,^ ^16^ ^,^ ^17^ BCP seems to affect the structure of the virion envelope of HSV-1, which is essential for adsorption or entry into the host cells.^18^

Both natural compounds have demonstrated anti-inflammatory, immunomodulatory, and wound healing properties that may be beneficial in herpes treatment.^19^ ^,^ ^20^ ^,^ ^21^ ^,^ ^22^ In addition to contributing to the control of inflammation associated with infection, their ability to support tissue repair may accelerate the recovery of virus-induced lesions, reduce the aesthetic and functional impact of skin damage, and help prevent complications, particularly in areas exposed to UV radiation.^23^

Nanoemulsions are colloidal dispersions composed of two immiscible phases, typically oil and water, stabilised by surfactants and co-surfactants, with droplet sizes generally ranging from 10 to 200 nm. Their nanometric size confers unique properties, including a large interfacial surface area and enhanced kinetic stability, which contribute to improved solubility, bioavailability, and controlled release of encapsulated bioactive compounds.^24^ These characteristics make nanoemulsions particularly suitable for the delivery of lipophilic natural compounds.

In this context, nanoencapsulation represents a promising strategy for compounds such as BCP and RSV, which present important pharmaceutical limitations. BCP is highly susceptible to air oxidation, leading to the formation of caryophyllene oxide, a less biologically active derivative, while its volatility further reduces therapeutic efficacy during storage.^25^ ^,^ ^26^ RSV, in turn, suffers from poor aqueous solubility, photoisomerisation from the active trans- to the inactive cis-form, and extensive first-pass metabolism, which severely limit its bioavailability.^27^ Nanoemulsions prepared by low-energy methods, such as spontaneous emulsification, provide a protective lipid environment that minimises degradation while enhancing interaction with biological membranes and facilitating cellular uptake.^24^ ^,^ ^28^ Therefore, nanoemulsions emerge as a rational strategy to improve the therapeutic performance of RSV and BCP.

Considering the variety of pharmacological activities described above and the already known safety profile of these compounds, the hypothesis of this study was to investigate whether both could act synergistically against HSV. Additionally, this study aimed to evaluate whether nanoencapsulation of these active compounds could improve their anti-herpetic activity.

## MATERIALS AND METHODS

*Materials* - BCP, RSV and Tween 80 were purchased from Sigma-Aldrich (St Louis, MO, USA). Medium-chain triglycerides (MCT; caprylic/capric triglycerides) were obtained from Croda (São Paulo, Brazil). Phosal^®^ 50 SA+, which is composed of more than 50% phosphatidylcholine in safflower seed oil, was donated by Lipoid (Ludwigshafen, Germany). Ethanol was obtained from Vetec Química Fina Ltda (Duque de Caxias, Brazil), and ultrapure water was obtained from a Milli-Q^®^ purification system. For biological activity assays, RSV and BCP were dissolved in dimethyl sulfoxide (DMSO; Merck).

Green monkey kidney fibroblasts (VERO - ATCC: CCL81) were grown in Eagle’s minimum essential medium (MEM; Gibco) and supplemented with 10% foetal bovine serum (FBS; Gibco, Thermo Fisher Scientific, Waltham, USA). Human gingival fibroblasts (HGF) were obtained by primary culture from three healthy patients who needed distal wedge procedures. The tissue removal protocol was approved by the Human Research Ethics Committee (CEPSH) at the Federal University of Santa Catarina (UFSC), under authorisation number 062, protocol number 21/09. The HGF cell line was cultured in Dulbecco’s Modified Eagle’s Medium (DMEM; Gibco, Thermo Fisher Scientific, Waltham, USA) with 10% FBS (Gibco, Thermo Fisher Scientific, Waltham, USA).

HSV-1 (KOS strain) stocks were donated from the Faculty of Pharmacy, University of Rennes, France. Viral titre was calculated as plaque-forming units (PFU).^29^

*Nanoemulsion preparation* - Nanoemulsions were prepared by using the spontaneous emulsification technique, a low-energy method, according to Singh et al.,^30^ and the formulation was based on Santos Porto et al.,^26^ with some modifications. The organic phase was composed of 0.5 g of BCP and/or 0.002 g of RSV, 0.25 g of medium-chain triglycerides (MTC), and 0.5 g of Phosal^®^ (PHO) in 25 mL of ethanol. For the aqueous phase, 0.1 g of Tween^®^ 80 was solubilised in 50 mL of Milli-Q water. A detailed composition of each formulation is presented in the Table I. The organic phase was slowly added to the aqueous phase using a syringe at room temperature. Finally, the organic solvent and excess water were removed under reduced pressure at 215 rpm and 32ºC, resulting in a final formulation volume of 10 mL.

**TABLE I:** Composition (%, w/w) of nanoemulsions with and without resveratrol (RSV) and β-caryophyllene (BCP)

Components	NE blank	NE BCP	NE RSV	NE BCP+RSV
BCP	-	5.0%	-	5.0%
RSV	-	-	0.02%	0.02%
MCT	2.5%	2.5%	2.5%	2.5%
PHO	5.0%	5.0%	5.0%	5.0%
Ethanol	25 mL	25 mL	25 mL	25 mL
Tween^®^ 80	1.0%	1.0%	1.0%	1.0%
Water	50 mL	50 mL	50 mL	50 mL

NE RSV: nanoemulsion containing resveratrol; NE BCP: nanoemulsion containing β-caryophyllene; NE RSV+BCP: nanoemulsion containing resveratrol and β-caryophyllene; NE blank: control nanoemulsion containing only excipients, without test compounds.

*Formulation characterisation: determination of particle size, polydispersity index, and surface charge* - Particle size and polydispersity index (PDI) were analysed using photon correlation spectroscopy at 25ºC with a laser beam angle of 90º. Zeta potential was determined through electrophoretic mobility measurements. All analyses were performed in triplicate using a Malvern Zetasizer Nano-ZS 90 instrument (Malvern Instruments, Malvern, UK). Colloidal suspensions were diluted in ultrapure water at a 1:10 (v/v) ratio for the measurements.

*Stability study: long-term stability* - For long-term stability tests, NEs were stored under refrigeration (4 ± 2ºC) and protected from light. During these conditions, measurements of particle size, polydispersity index and zeta potential were carried out after 0, 15, 30, 60, and 90 days of storage. All samples were analysed in triplicate and results were expressed as mean ± standard deviation (SD).

*Antiviral activity: in vitro cytotoxicity assay* - The cytotoxicity assay was carried out using the sulforhodamine B colorimetric assay.^31^ Briefly, 96-well plates previously prepared with VERO cell suspensions, at a density of 2.5x10^4^ cells/well, were cultured in MEM medium, supplemented with 10% FBS, and maintained at 37ºC in a humidified atmosphere containing 5% CO_2_ for 24 h. After 24 h, 100 μL of test materials, at different concentrations, was added to each well and plates were incubated for a further 48 h under culture conditions. RSV was tested at concentrations from 0.03 to 15.62 µM, BCP from 7.45 to 3812.5 µM, and the RSV+BCP combination was tested at the same concentrations in a fixed ratio (1:244). After the treatment time, the cells were fixed by adding a 10% (w/v) TCA aqueous solution, and the plate was incubated for 1 h at 4ºC. Subsequently, the fixed cells were stained with 100 μL of the acidic sulforhodamine B solution (0.057% w/v) for 30 min and the dye bound to the proteins was extracted with the addition of a 10 mM Tris Base buffer solution (pH 10.5). Finally, the absorbance values were measured in a spectrophotometer (Spectra MD2, Molecular Devices, Sunnyvale, CA, USA) at 510 nm. All assays were performed in triplicate. The 50% cytotoxic concentration (CC_50_), which represents the concentration of test compound required to reduce cell viability by 50%, was calculated by nonlinear regression analysis.

*Viral plaque number reduction assay* - VERO cells (2.5 x 10^5^/well) were growth in MEM supplemented with 10% FBS in 24-well plates under culture conditions (37ºC and 5% CO_2_). After 24 h, the medium was aspirated, and the cell monolayer was infected with 400 μL of viral suspension (100 PFU/well). After 1 h of adsorption at 37ºC and 5% CO_2_, 500 μL of MEM containing 1.5% carboxymethylcellulose (CMC), with or without different concentrations of the samples or acyclovir 5 µM (positive control) was added. The plates were incubated for 48 h at 37ºC and 5% CO_2_. After incubation, the cells were stained with a naphthol blue-black solution for 20 min at room temperature under agitation, and the plaques were counted using a stereoscopic microscope. The percentage of viral replication inhibition for each sample relative to viral controls was calculated and plotted on a graph. IC_50_ values were determined through nonlinear regression analysis, representing the average of three independent experiments.

*Wound-healing assay and UV protection capacity: cytotoxicity assay* - The cytotoxicity of NE RSV, NE BCP, and NE RSV+BCP was assessed using the MTT colorimetric assay.^32^ Briefly, 96-well plates were seeded with HGF cell suspensions at a density of 1 × 10⁴ cells/well. The cells were cultured in DMEM supplemented with 10% FBS and maintained at 37ºC in a humidified atmosphere containing 5% CO₂ for 24 h. After the initial incubation period, 100 µL of the test materials, at varying concentrations, was added to each well, and the plates were incubated for an additional 48 h under the same culture conditions.

NE RSV was tested at concentrations ranging from 0.03 to 15.62 µM, NE BCP from 7.45 to 3812.5 µM, and the NE RSV+BCP combination was tested at the same concentrations in a fixed ratio (1:244). The NE Blank was tested at the same dilutions and volumes as the NE RSV+BCP. Following the treatment period, the supernatant was removed, and 100 µL of a 1 mg/mL MTT solution in DMEM was added to each well. The plates were then incubated for 4 h at 37ºC. Subsequently, 100 µL of DMSO was added to dissolve the formazan crystals, and the absorbance was measured using a spectrophotometer (Spectra MD2, Molecular Devices, Sunnyvale, CA, USA) at a wavelength of 540 nm. All experiments were performed in triplicate.

The 50% cytotoxic concentration (CC₅₀), defined as the concentration of the test material required to reduce cell viability by 50%, was determined using nonlinear regression analysis.

*Scratch assay* - Cell migration and proliferation activity was evaluated using a cell migration assay (scratch assay), as described by Suarez-Arnedo et al.^33^ Initially, HGF cells (2.5 × 10⁵ cells/well) were cultured in 12-well plates and incubated at 37ºC and 5% CO₂ for 24 h until reaching confluence. After the formation of the cell monolayer, a linear artificial wound was created using the tip of a sterile micropipette. Subsequently, samples at different concentrations were added to the cells and incubated at 37ºC and 5% CO₂. Representative images of the wounded areas were captured at 0, 24, and 48 h to estimate relative cell migration. Image analysis was performed using ImageJ software. The percentage of wound closure at each experimental time point was calculated according to the following equation:


$$Wound\;closure\;\left(\%\right)=\frac{\left(Initial\;wound\;area-wound\;area\;after\;“n”\;hours\right)}{\left(Initial\;wound\;area\right)}x\;100\%$$

All assays were performed in triplicate, and the data were expressed as the mean of three independent experiments with 95% confidence intervals (CI).

*Ultraviolet Crosslinker (UVC) irradiation and drug administration* - HGF cells were seeded in 12-well plates at a density of 2 x 10⁵ cells/well and incubated for 24 h. After this period, the culture medium was removed and replaced with fresh medium containing varying concentrations of NE RSV, NE BCP, RSV, and BCP. The cells were then treated for 2 h or simultaneously exposed to UVC radiation. Irradiation was performed using the UVC 500 Ultraviolet Crosslinker (Hoefer, USA) at a dose of 50 mJ/cm² for 30 min. Subsequently, cell viability was assessed using the MTT assay, as described in Section 2.6.1. The control group was subjected to the same experimental conditions but without UVC radiation exposure.

*Statistical analysis* - Data are presented as mean ± SD from three independent experiments. CC₅₀ and IC₅₀ values were estimated by nonlinear regression of concentration-response curves using GraphPad Prism 8 software (GraphPad, Boston, USA).

## RESULTS

*Nanoemulsion characterisation* - Regardless of the composition, the colloidal systems showed a reduced particle size and a homogeneous size distribution, as PDI values close to 0.2 were found. A negative surface charge was found for the particles, which can be associated with free fatty acids or lipids from the MCT or Phosal^®^. The presence of BCP was shown to affect this parameter, reducing the charge density (Table II).

**TABLE II:** Size, polydispersity index (PDI), and zeta potential of nanoemulsions. Results are expressed as mean ± standard deviation (SD) (n = 3)

Formulation	Size (nm)	PDI	Zeta potential (mV)
NE RSV	217.4 ± 7.48	0.187 ± 0.015	-18.60 ± 3.25
NE BCP	205.1 ± 5.32	0.187 ± 0.014	-9.31 ± 0.51
NE RSV+BCP	205.4 ± 8.89	0.201 ± 0.006	-11.41 ± 3.53
NE Blank	196.4 ± 12.82	0.185 ± 0.013	-19.70 ± 3.37

NE RSV: nanoemulsion containing resveratrol; NE BCP: nanoemulsion containing β-caryophyllene; NE RSV+BCP: nanoemulsion containing resveratrol and β-caryophyllene; NE blank: control nanoemulsion containing only excipients, without test compounds.

*Stability studies* - The stability of the nanoemulsions was monitored for up to 90 days after preparation. Size, zeta potential and PDI remained stable during this period (Fig. 1). No microbial growth was macroscopically observed (data not shown).

**Fig. 1:**
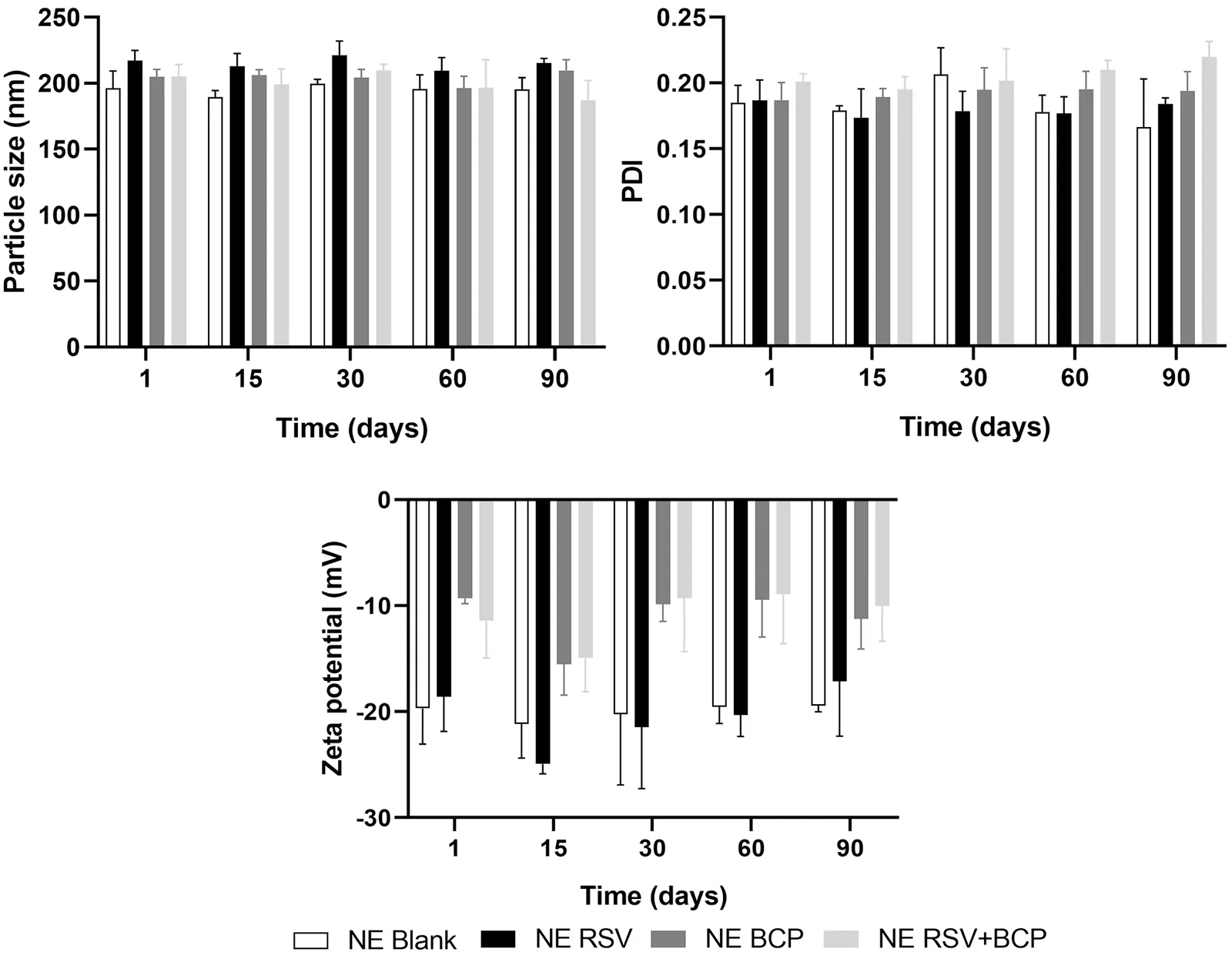
particle size, zeta potential, and polydispersity index (PDI) of nanoemulsions stored at 4 ± 2ºC for 90 days (n = 3).

*Antiviral activity* - The cytotoxicity results of free compounds and nanoemulsions are summarised in Table III.

**TABLE III:** Cytotoxicity of free compounds and nanoemulsions in VERO cells determined by the sulforhodamine B assay

Samples	CC_50_ (μM)
RSV	> 15.00
BCP	1423.00 ± 256.9
RSV+BCP	4.24 ± 0.66 RSV+1034.00 ± 160.0 BCP
NE RSV	> 15.00
NE BCP	365.1 ± 12.1
NE RSV+BCP	17.53 ± 2.2 RSV+4277.00 ± 537.3 BCP

RSV: resveratrol; BCP: β-caryophyllene; NE RSV: nanoemulsion containing resveratrol; NE BCP: nanoemulsion containing β-caryophyllene; NE RSV+BCP: nanoemulsion containing both compounds. CC₅₀ represents the concentration required to reduce cell viability by 50%. Values are expressed in µM and represent the mean ± standard deviation (SD) of three independent experiments (n = 3). These results were used to guide the selection of non-cytotoxic concentrations for subsequent antiviral assays.

RSV did not exhibit cytotoxicity at the evaluated concentrations (0.03-15.63 µM), as cell viability values remained above 75%. Its incorporation into nanoemulsions improved biocompatibility, resulting in cell viability percentages close to 100%. In contrast, BCP showed the opposite effect. Nanoencapsulation increased its toxicity, with a significant reduction in CC₅₀ values, from 1423 µM for free BCP to 365.1 µM for NE BCP. An inverse trend was observed for the combination of active ingredients. The RSV+BCP system exhibited CC₅₀ values of 4.24 µM for RSV and 1034 µM for BCP, whereas, after the preparation of the RSV+BCP NE, the CC₅₀ value increased to 17.53 µM for RSV and 4277 µM for BCP, indicating a reduction in cytotoxicity*.* The blank formulation (without active compounds) did not exhibit cytotoxicity.

In antiviral activity assays with isolated compounds (Fig. 2), RSV demonstrated greater potency than BCP. Indeed, lower concentrations of RSV were required to achieve an inhibitory effect against HSV-1. At non-cytotoxic concentrations (up to 1423 µM for BCP), the combination of RSV and BCP did not result in enhanced anti-herpetic activity compared to assays performed with the isolated compounds. At 953 µM, BCP was more active than RSV; however, this result should be interpreted with caution, as BCP exhibited some toxicity at these concentrations.

**Fig. 2:**
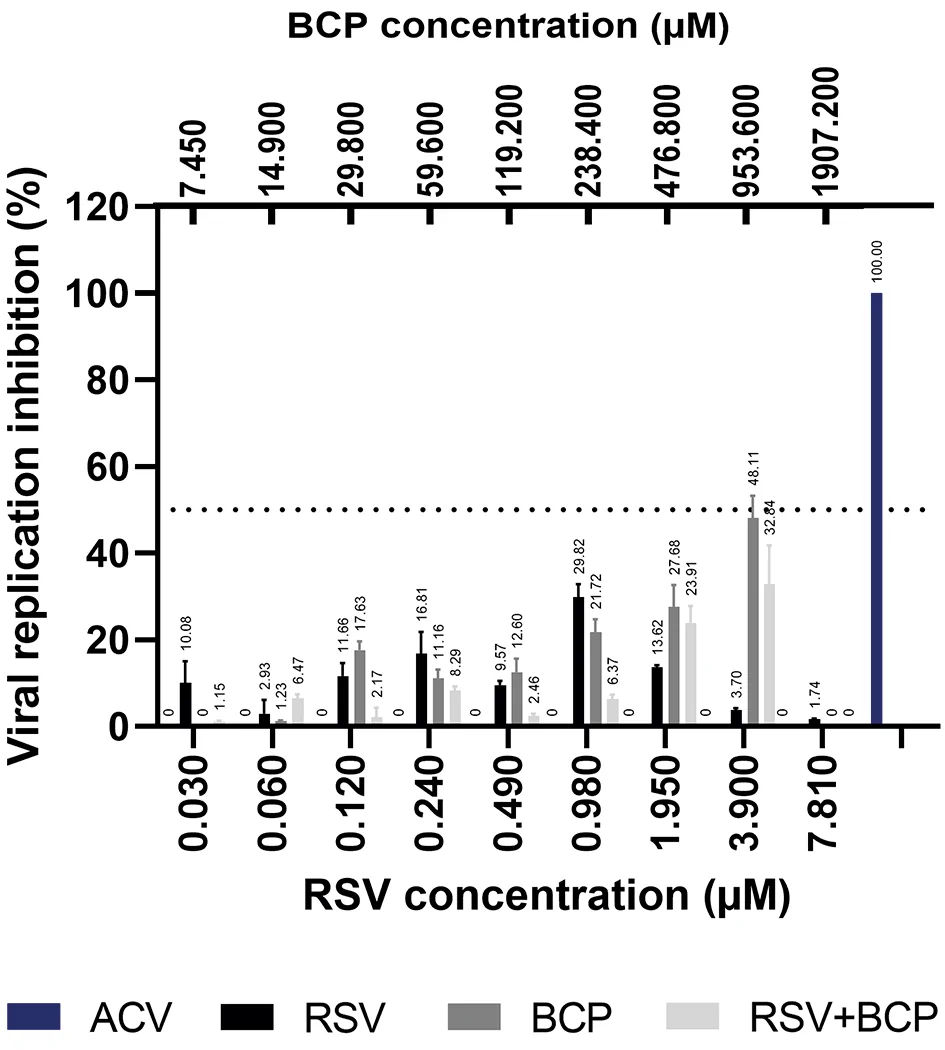
effect of resveratrol (RSV), β-caryophyllene (BCP), and their combination (RSV+BCP) on Herpes Simplex virus type 1 (HSV-1) infection in VERO cells determined by viral plaque reduction assay. Antiviral activity was expressed as percentage of viral inhibition relative to the untreated infected control. The horizontal dashed line indicates 50% viral inhibition. Data are presented as mean ± standard deviation (SD) of three independent experiments (n = 3).

The concentration ranges evaluated in the antiviral assays were selected based on the CC₅₀ values obtained in VERO cells to ensure that all treatments were tested within a non-cytotoxic concentration range. The concentration-response curves used for CC₅₀ and IC₅₀ determination are provided in the Supplementary data (Figs 1-4).

After nanoencapsulation, NE RSV exhibited weak inhibitory activity against HSV-1, similar to the blank formulation, while NE BCP showed inhibition percentages close to 50%, with an IC₅₀ value of 115.80 µM. On the other hand, nanoemulsions containing both RSV and BCP demonstrated enhanced antiviral activity, achieving inhibition percentages above 70%, with an IC₅₀ value of 0.11 + 26.83 µM and a selectivity index (SI) of 159.36 (Fig. 3).

**Fig. 3:**
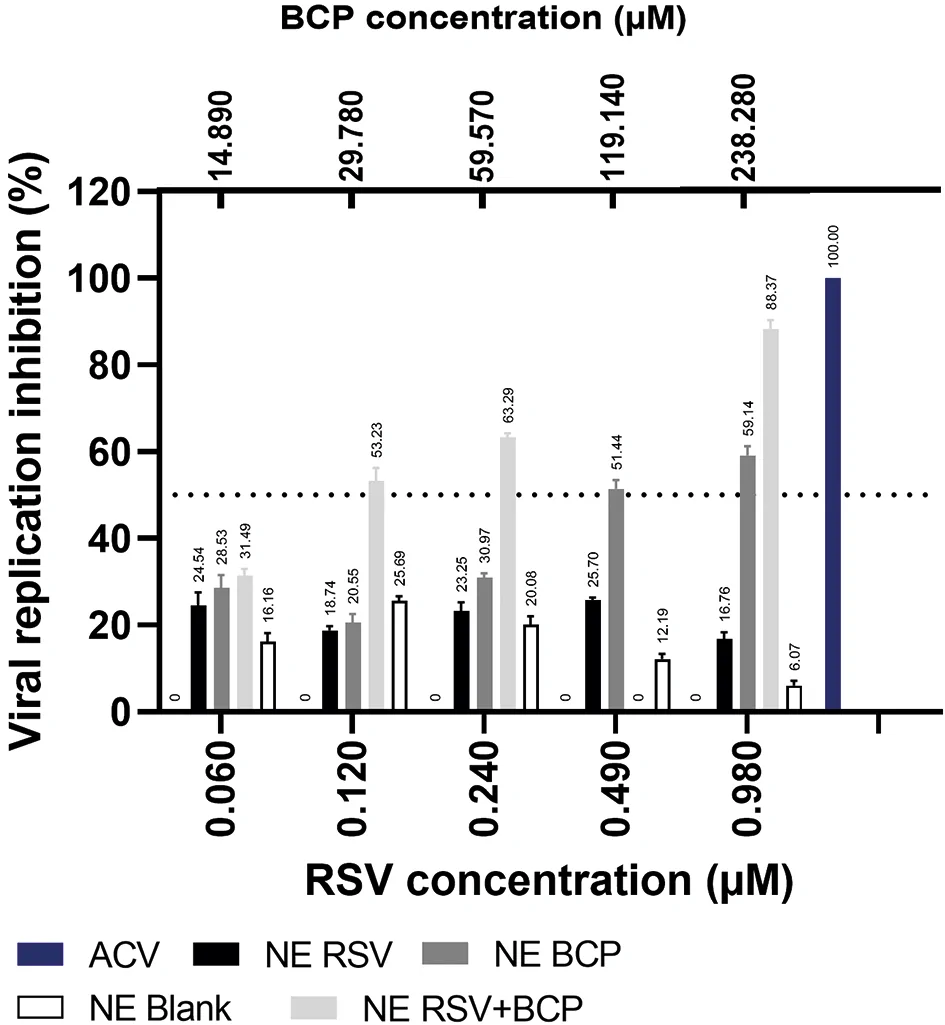
effect of nanoemulsions containing resveratrol (NE RSV), β-caryophyllene (NE BCP), and their combination (NE RSV+BCP) on Herpes Simplex virus type 1 (HSV-1) infection in VERO cells determined by viral plaque reduction assay. Antiviral activity was expressed as percentage of viral inhibition relative to the untreated infected control. The horizontal dashed line indicates 50% viral inhibition. Data are presented as mean ± standard deviation (SD) of three independent experiments (n = 3).

*Wound-healing assay and UV protection capacity: cytotoxicity assay* - As shown in Table IV, the RSV nanoemulsions (NE RSV) demonstrated a higher CC_50_ value compared to that observed in the VERO cell line. In contrast, the CC_50_ value for the BCP nanoemulsion (NE BCP) remained very similar. However, for the combined RSV+BCP nanoemulsion (NE RSV+BCP), the CC_50_ value was approximately 12 times lower compared to that observed in the VERO cell line.

**TABLE IV:** Cytotoxicity of nanoemulsions in human gingival fibroblast (HGF) cells determined by the MTT assay

Cell line	NE RSV	NE BCP	NE RSV+BCP
	CC_50_ μM	CC_50_ μM	CC_50_ μM
HGF	122.9 ± 4.98	359.8 ± 48.84	1.37 ± 0.38 RSV+335.2 ± 47.25 BCP

NE RSV: nanoemulsion containing resveratrol; NE BCP: nanoemulsion containing β-caryophyllene; NE RSV+BCP: nanoemulsion containing resveratrol and β-caryophyllene; CC_50_: 50% cytotoxic concentration (μM). Values are expressed in µM and represent the mean ± standard deviation (SD) of three independent experiments (n = 3).

*Scratch assay* - Based on the results of the antiviral activity evaluation, concentrations of 0.98 µM NE RSV and 238.00 µM NE BCP were found to inhibit viral replication. Given these findings, preliminary experiments were conducted to investigate the potential effects of NE RSV, NE BCP, and NE RSV+BCP on the cellular wound healing process. The results demonstrated that, 24 h after wound induction, cells treated with NE BCP and NE RSV+BCP appeared detached, suggesting a possible interference with cell adhesion and migration, critical steps for wound closure. In contrast, treatment with NE RSV resulted in a slightly higher wound closure rate compared to the cell control, with percentages of 47.47% and 43.57%, respectively. After 48 h, the control group exhibited a wound closure rate of 76.25%, while the NE RSV treatment achieved 68.80%, indicating that although NE RSV promoted a more pronounced initial effect, the control group showed more consistent recovery over time. The NE Blank was also evaluated under the same experimental conditions and showed wound closure percentages of 46.06% and 58.95% after 24 and 48 h, respectively. Representative images of all experimental groups, including the blank formulation, are provided in the Supplementary data (Fig. 5).

To further explore these observations, three distinct concentrations of NE RSV (40, 20, and 10 µM) and NE BCP (125.00, 62.5, and 31.25 µM) were tested to evaluate their potential effects on accelerating the cellular healing process. The results demonstrated that neither NE RSV nor NE BCP promoted wound healing compared to the cell control, as evidenced in Figs 4-5. Notably, the highest concentration of NE RSV (40 µM) resulted in a significant reduction in wound closure compared to the control group. No statistically significant differences were observed among the remaining concentrations evaluated, suggesting that variations in dosage within the tested range did not substantially influence the tissue repair process.

**Fig. 4:**
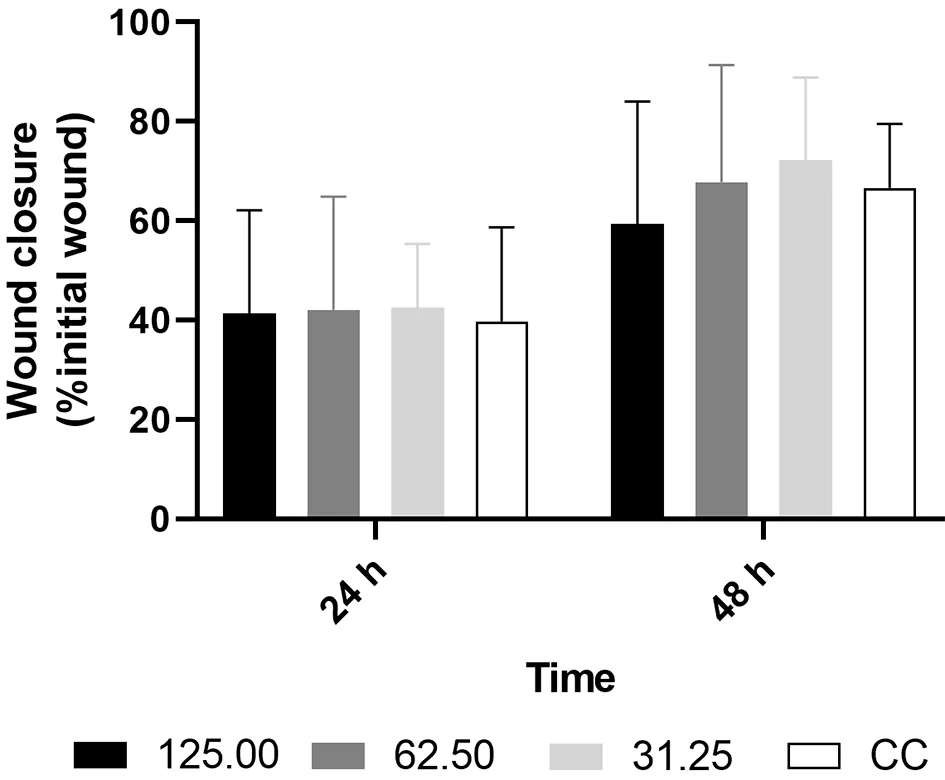
migration assay: wound closure percentage for nanoemulsions containing β-caryophyllene (NE BCP) (125.00 μM, 62.5 μM, and 31.25 μM) and untreated cells (CC) at 24 h and 48 hs. The tests were conducted on human gingival fibroblast (HGF) cells, and the results represent the average of three independent experiments.

**Fig. 5:**
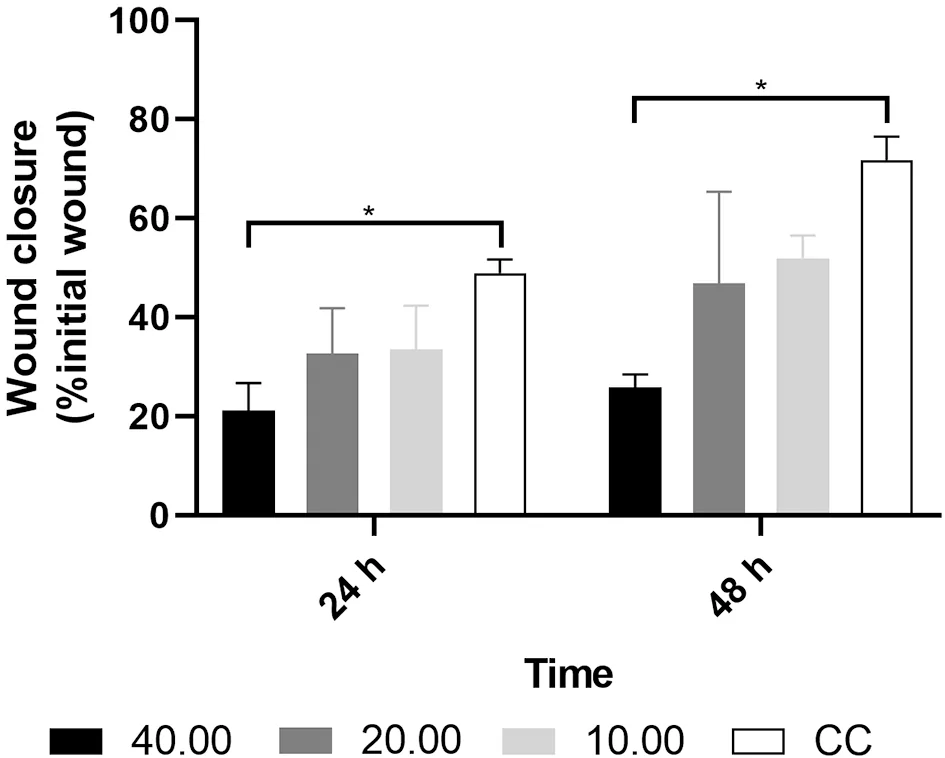
migration assay: wound closure percentage for nanoemulsions containing resveratrol (NE RSV) (40 μM, 20 μM, and 10 μM) and untreated cells (CC) at 24 h and 48 h. The tests were conducted on human gingival fibroblast (HGF) cells, and the results represent the average of three independent experiments. *Significant values (p < 0.05); one-way ANOVA followed by Tukey’s Post Hoc Test.

*UVC irradiation and drug administration* - Based on the antiviral activity results, the potential protective effects of NE RSV, NE BCP, and the combined NE RSV+BCP were initially assessed at concentrations of 0.98 μM, 238.00 μM, and their combination. As presented in Table V, NE RSV-treated cells exposed to UV irradiation showed viability levels comparable to those of the UV-exposed untreated cells, in contrast to cells subjected to simultaneous treatment and UV exposure. For the other formulations and experimental conditions, cell viability values remained largely comparable across the different treatment modalities.

**TABLE V:** Cell viability (%) after simultaneous treatment or 2 h pretreatment with nanoemulsion containing resveratrol (NE-RSV) (0.98 μM), nanoemulsion containing β-caryophyllene (NE-BCP) (238 μM), and nanoemulsion containing resveratrol and β-caryophyllene (NE-RSV+BCP) (0.98 + 238 μM) under UV-exposed and non-exposed conditions

	Samples	Simultaneous treatment	2 h pretreatment
		% cell viability	% cell viability
Exposed to UV	NE RSV	10.01 ± 3.80	54.92 ± 6.29
	NE BCP	17.92 ± 2.50	16.10 ± 1.77
	NE RSV+BCP	9.04 ± 2.19	8.07 ± 3.71
	Untreated cells	52.03 ± 1.48	55.89 ± 2.34
Not exposed to UV	NE RSV	77.90 ± 7.72	77.95 ± 2.81
	NE BCP	66.26 ± 6.84	63.23 ± 6.23
	NE RSV+BCP	52.49 ± 3.05	53.10 ± 2.64
	Untreated cells	101.41 ± 2.00	100.00 ± 0.71

Values are expressed in µM and represent the mean ± standard deviation (SD) of three independent experiments (n = 3).

**TABLE VI:** Cell viability (%) after simultaneous treatment or 2 h pretreatment with nanoemulsion containing resveratrol (NE-RSV) and nanoemulsion containing β-caryophyllene (NE-BCP) under UV-exposed and non-exposed conditions

	Samples	Concentration (μM)	Simultaneous treatment	2 h pretreatment
			% cell viability	% cell viability
Exposed to UV	NE RSV	40.00	46.67 ± 2.26	48.79 ± 6.49
		20.00	50.73 ± 5.33	32.71 ± 6.50
		10.00	24.72 ± 0.42	27.40 ± 3.34
	NE BCP	125.00	20.66 ± 1.10	21.54 ± 2.25
		62.50	16.54 ± 1.57	24.66 ± 5.54
		31.25	12.73 ± 2.10	27.60 ± 6.67
	Untreated cells	-	52.03 ±1.48	55.89 ± 2.34
Not exposed to UV	NE RSV	40.00	63.82 ± 3.49	65.40 ± 3.15
		20.00	70.81 ± 3.48	74.31 ± 4.19
		10.00	75.33 ± 1.05	75.53 ± 5.32
	NE BCP	125.00	76.04 ± 0.97	77.34 ±5.52
		62.50	80.68 ± 2.61	87.88 ± 4.74
		31.25	82.49 ± 1.54	87.78 ± 0.59
	Untreated cells	-	101.41 ± 2.00	100.00 ± 1.13

Values are expressed in µM and represent the mean ± standard deviation (SD) of three independent experiments (n = 3).

Given these observations, additional assays were carried out using a concentration range of NE RSV (40, 20, and 10 μM) and NE BCP (125.00, 62.50, and 31.25 μM). The data indicate that, even at the higher concentrations tested, cell viability remained lower than that of the corresponding experimental untreated group (Table VI). Since no improvement in cell viability was observed for NE RSV+BCP under the initial experimental conditions, subsequent experiments were designed to evaluate whether different concentration ranges of the individual nanoemulsions (NE RSV and NE BCP) could provide protective effects against UVC-induced damage. As neither formulation showed beneficial effects in these additional assays, further evaluation of the combined nanoemulsion was not performed.

## DISCUSSION

In this work, we investigated the therapeutic potential of nanoemulsions containing RSV and BCP for the treatment of HSV-1 infections. The developed formulations presented excellent physicochemical characteristics, with low PDI values (~0.2) indicating homogeneous particle size distribution, essential for colloidal stability.^34^ ^,^ ^35^ The negative surface charge (-18.60 mV for NE RSV and -19.70 mV for NE Blank) probably results from ionised fatty acids present in MCT and Phosal^®^, and the addition of BCP significantly reduced this zeta potential to -9.31 mV (NE BCP) and -11.41 mV (NE RSV+BCP), suggesting specific interactions with surfactant compounds that modify the nanoparticle interface.^34^ ^,^ ^35^ ^,^ ^36^ ^,^ ^37^

The stability of the formulations was maintained for 90 days under refrigeration, which can be attributed both to the intrinsic antimicrobial properties of the encapsulated active compounds^26^ ^,^ ^38^ and to the presence of the surfactant Tween^®^ 80, which contributed to the stability of the system by reducing droplet aggregation through hydrophobic interactions.^39^

From a biological point of view, RSV maintained an excellent safety profile, with cell viability above 75% at all concentrations tested (0.03-15.63 µM), corroborating the findings of Docherty et al.,^40^ Huang et al.^41^ and Zangooei et al.^42^ Docherty et al.^40^ demonstrated that concentrations of 25 μg/mL (~109 μM) inhibited 95% of viral replication in VERO and MRC-5 cells, while 50 μg/mL (~218 μM) completely abolished replication after 72 h of treatment, through specific inhibition of the expression of ICP-4 proteins, essential for viral replication. Complementing these findings, Leyton et al.^43^ observed in hippocampal neurons infected with HSV-1 that treatment with RSV (100 μM) reduced viral protein levels by 50%, in addition to decreasing neurodegenerative markers (TauC3 and p-Tau), suggesting an additional neuroprotective effect.^43^

In contrast, BCP showed distinct behaviour after nanoencapsulation, with increased toxicity (reduction in CC₅₀ from 1423 μM to 365.1 μM), in line with the results of Dahham et al. in several cell lines.^44^ However, its antiviral activity was preserved, with IC₅₀ of 115.80 μM in the nanoencapsulated form, possibly due to the protection against oxidation provided by the nanoemulsion system.^25^ This activity is in agreement with the studies of Schnitzler et al.^45^ and Astani et al.,^18^ which demonstrated the ability of BCP to damage the viral envelope, significantly reducing the infectivity of HSV-1.^18^ ^,^ ^45^

The most promising result was observed in the nanoencapsulated combination RSV+BCP, which showed remarkable antiviral synergy (IC₅₀ of 0.11 + 26.83 μM) with a high selectivity index (159.36). This synergy appears to result from the combination of two complementary mechanisms: the action of BCP in the extracellular viral inactivation, through damage to the viral envelope, and intracellular inhibition of viral replication by RSV, via suppression of the expression of essential viral proteins.^18^ ^,^ ^40^ ^,^ ^45^ Nanoencapsulation enhanced this synergy, probably by maintaining the ideal ratio between the compounds during cellular delivery and protection against degradation.

The association between HSV infection and impaired wound healing has been widely reported, as viral replication can interfere with cellular processes involved in tissue repair, leading to delayed wound closure and increased tissue damage.^3^ ^,^ ^46^ This condition may be further exacerbated by UV-induced oxidative stress, which is also known to promote cellular damage and contribute to HSV reactivation.^47^

From a therapeutic perspective, these factors highlight the importance of developing strategies that combine antiviral activity with tissue-protective effects. Therefore, evaluating the potential of nanoemulsions not only in inhibiting viral replication but also in supporting tissue repair and protecting against environmental stressors becomes particularly relevant.

In addition to the antiviral activity, the healing and photoprotective potential of the nanoformulations was investigated. For this evaluation, HGF were selected as a biological model, and the cytotoxicity of the nanoemulsions was initially evaluated. In particular, the increase in the CC_50_ value of NE RSV suggests a greater biocompatibility of resveratrol when formulated in nanoemulsions, while the stability of the CC_50_ observed in NE BCP indicates that the nanoencapsulation maintained the characteristic cytotoxic profile of this compound. The most relevant finding was the significant 12-fold reduction in the CC₅₀ of the combined formulation NE RSV+BCP, suggesting a potential combinatorial effect, likely associated with enhanced cellular interaction and overlapping mechanisms of action at the cellular level, as commonly observed in nanoemulsion-based delivery systems.^24^ ^,^ ^38^

In the wound healing studies, NE RSV showed a slightly higher wound closure percentage at 24 h (47.47% versus 43.57% in the control). However, when different concentrations were evaluated, the highest concentration tested (40 µM) resulted in a statistically significant reduction in wound closure compared to the control group. At 48 h, the control group showed more consistent recovery (76.25%) compared to NE RSV (68.80%). In addition, the NE Blank showed wound closure percentages of 46.06% and 58.95% after 24 and 48 h, respectively, and did not exhibit the pronounced loss of cell adhesion observed for NE BCP and NE RSV+BCP. These findings suggest that, under the evaluated experimental conditions, NE RSV did not promote wound healing and may even impair the wound closure process at higher concentrations. This observation is consistent with the known antifibrotic activity of RSV, which, as demonstrated by Bai et al.,^48^ Ishikawa et al.^49^ and Rahal et al.,^50^ acts through modulation of the TGF-β pathway, a mechanism that may influence cellular migration and tissue remodeling.^51^ Interestingly, our data partially contrast with the findings of Pang et al.^52^ and Smith et al.,^53^ who observed concentration-dependent effects of RSV on wound healing in different cellular models. This apparent contradiction, also noted by the authors themselves, reinforces the importance of factors such as cell type and concentration in the response to RSV. Overall, the evaluated nanoemulsions did not promote wound healing, suggesting that nanoencapsulation may modulate the biological activity of RSV under the experimental conditions investigated.

As for BCP, our results diverge from those reported by KK Aathira et al.,^54^ who observed an acceleration of cell migration (91.34% in 48 h with 145.3 µM) using free BCP. Therefore, this comparison should be interpreted with caution, as nanoencapsulation may significantly alter the interaction of the compound with cells and the extracellular matrix. The cell detachment induced by NE BCP and NE RSV+BCP in our study suggests that the nanoformulation can fundamentally alter the interaction of the compound with the extracellular matrix. It is plausible that the activation of the CB2 receptor by BCP, well documented by Koyama et al.^20^ as a pro-healing mechanism, is being affected by the nanoencapsulated delivery system.^55^

Regarding photoprotective properties, the results of the present study reveal significant differences in the protective effects against UVC radiation between RSV and BCP nanoemulsions and their free counterparts described in the literature. While Chan et al.^56^ demonstrated a clear protective effect of free RSV (10 µM) in ARPE19 cells, maintaining 75% viability after UVA exposure, a result also found in the work of Subedi et al.,^57^ RSV nanoemulsions only preserved viability at levels similar to untreated controls. This difference becomes evident when compared with the data of Cui et al.,^58^ who observed increases of up to 19.45% in the viability of HaCaT cells treated with free RSV in the same concentration range used in our study.

The apparent reduction in the protective efficacy of nanoformulations can be attributed to several factors. First, nanoencapsulation can alter the release kinetics of RSV, delaying or reducing its intracellular availability at the time of exposure to UVC radiation.^59^ Furthermore, the nanostructured formulation itself may interfere with RSV mechanisms.^60^ ^,^ ^61^ Finally, differences in experimental protocols, particularly in the time of exposure to UV radiation, the type of radiation and distance may contribute to the observed differences.

In the case of BCP, the absence of a protective effect in our nanoemulsions, even at relatively high concentrations (up to 238 µM), contrasts with studies demonstrating significant biological activities of this compound in other experimental models, including effects on cell proliferation and wound healing.^20^ ^,^ ^55^ This suggests that nanoencapsulation may be preventing BCP from accessing its molecular targets or that the mechanisms of protection against UVC damage require free forms of the compound.

*Final consideration* - In conclusion, this study demonstrated the potential of nanoemulsions containing RSV and BCP as a novel approach for the treatment of HSV-1 infections. The nanoemulsions exhibited significant antiviral activity, particularly when RSV and BCP were combined. However, the increased cytotoxicity of BCP following nanoencapsulation warrants further investigation to optimise the formulation for clinical use. Although the nanoemulsions did not show significant improvement in wound healing or protective effects against UV radiation exposure, their stability and antiviral efficacy reinforce their potential for topical application. Both nanoemulsions displayed monodispersion and maintained appropriate particle size and stability for up to 90 days when stored at 4ºC. These findings suggest that additional studies are needed to elucidate the underlying mechanisms of action, optimise the formulation for enhanced wound-healing properties, and evaluate its safety and efficacy in vivo.

## SUPPLEMENTARY MATERIALS

Supplementary material

## Data Availability

After publication the data will be available on demand to corresponding author.
