# Supplementary material for: Nanoemulsions loaded with ß-caryophyllene and resveratrol for herpes infections
Source: Mem Inst Oswaldo Cruz. 2026 Oct 12;121:e250333. doi: 10.1590/0074-02760250333 (PMC13660975; doi:10.1590/0074-02760250333)
Supplement: Supplementary file 1 [file 1678-8060-mioc-121-e250333-s1.pdf]

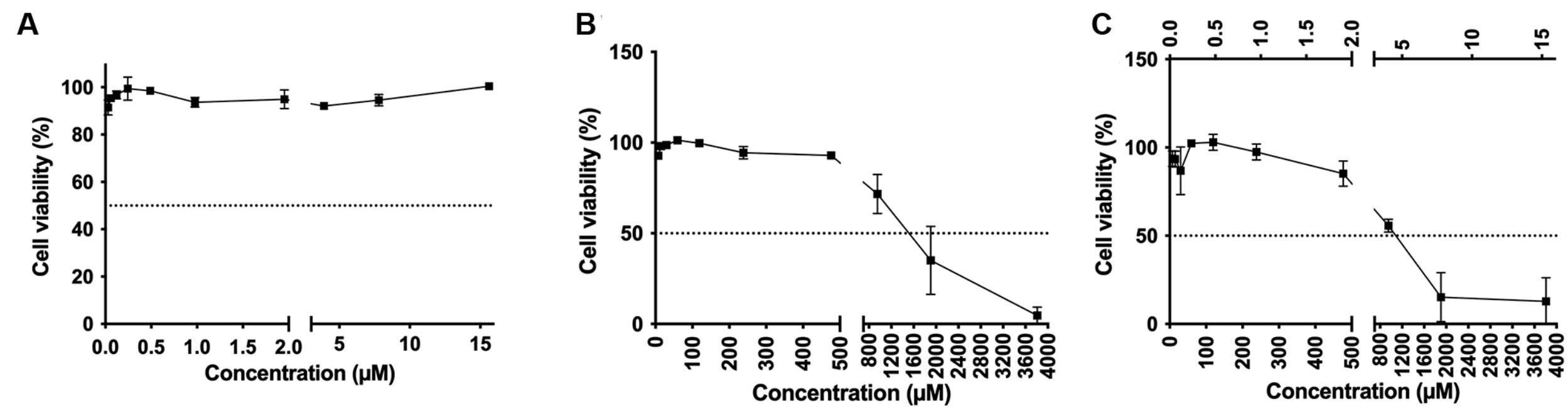

Fig. 1: concentration-response curves used for CC_{50} determination of RSV, BCP, and RSV+BCP in VERO cells. VERO cells were exposed to increasing concentrations of RSV (A), BCP (B), or RSV+BCP (C), and cell viability was assessed by the SRB assay. Data are expressed as mean ± SD of independent experiments. CC_{50} values were obtained by nonlinear regression analysis.

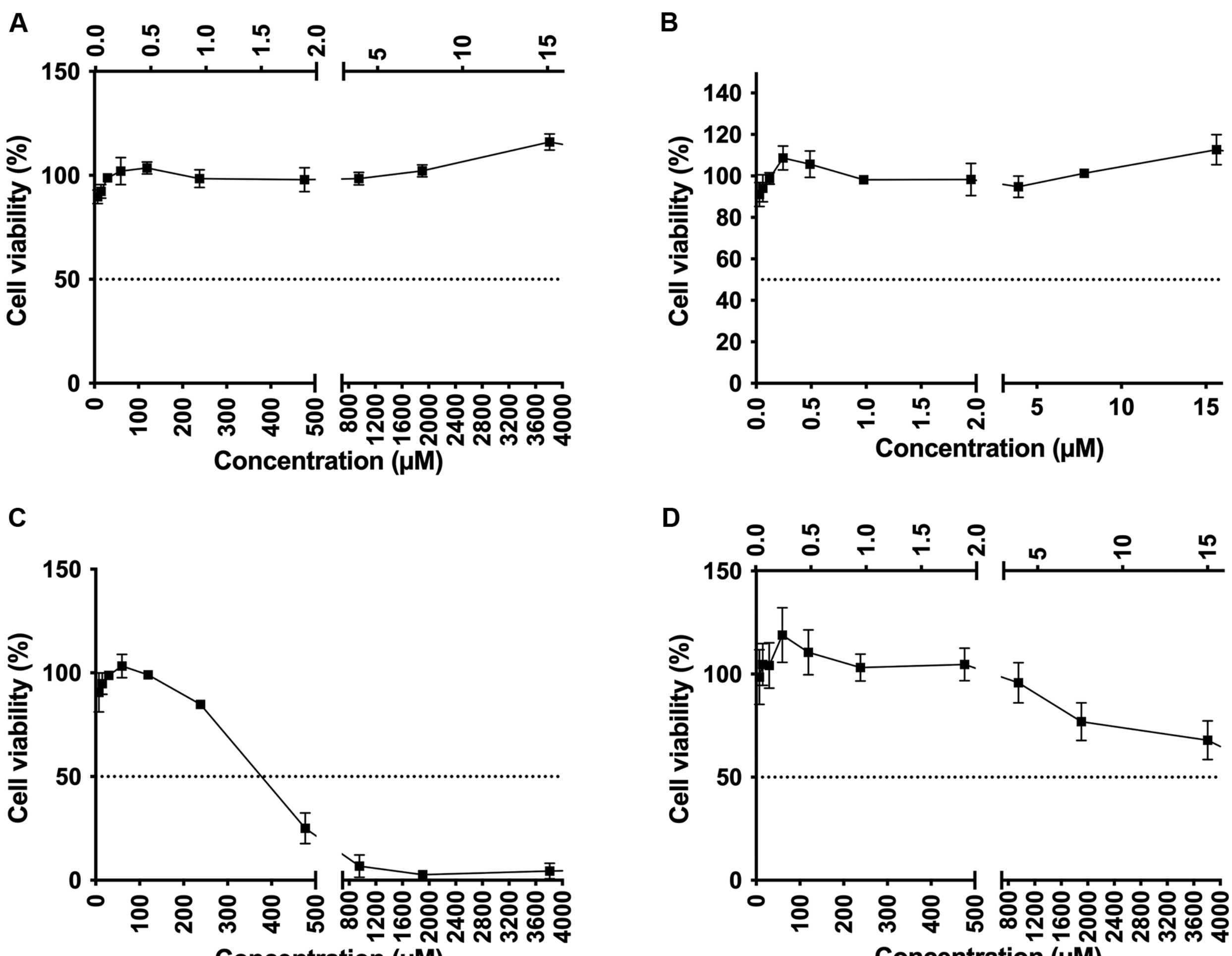

Fig. 2: concentration-response curves for the determination of CC_{50} values of NE Blank, NE RSV, NE BCP, and NE RSV+BCP in VERO cells. VERO cells were exposed to increasing concentrations of NE Blank (A), NE RSV (B), NE BCP (C), or NE RSV+BCP (D), and cell viability was assessed by the SRB assay. Data are expressed as mean ± SD of independent experiments. CC_{50} values were obtained by nonlinear regression analysis.

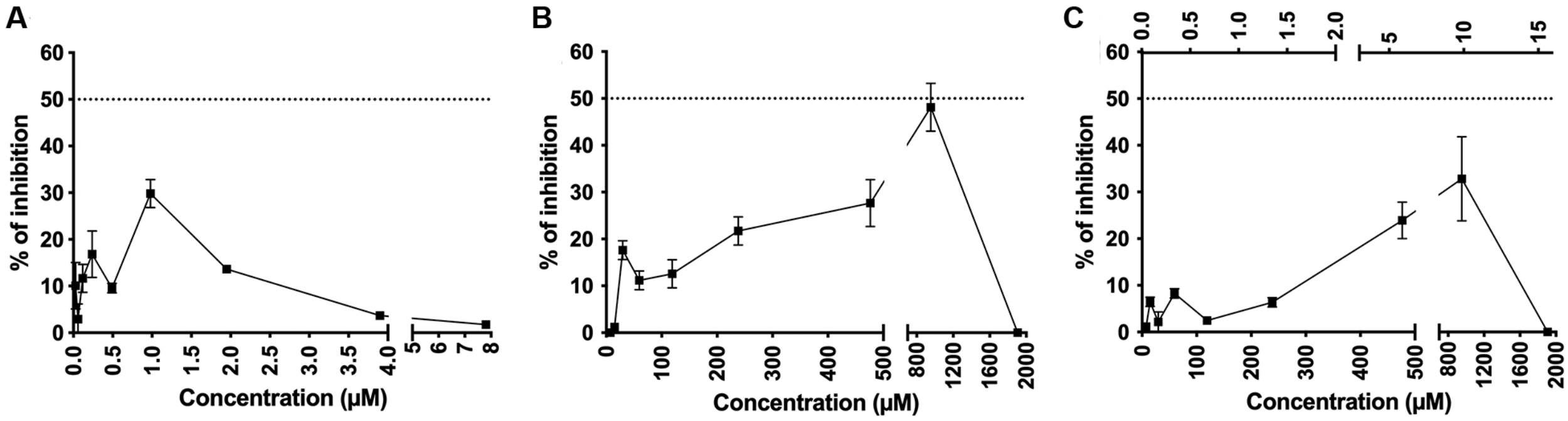

Fig. 3: dose-response curves for the determination of IC_{50} values of RSV, BCP, and RSV+BCP against HSV-1 (KOS strain). HSV-1 (KOS strain)-infected VERO cells were treated with increasing concentrations of RSV (A), BCP (B), or RSV+BCP (C), and antiviral activity was evaluated by plaque reduction assay. Data are expressed as mean ± SD of independent experiments. IC_{50} values were obtained by nonlinear regression analysis.

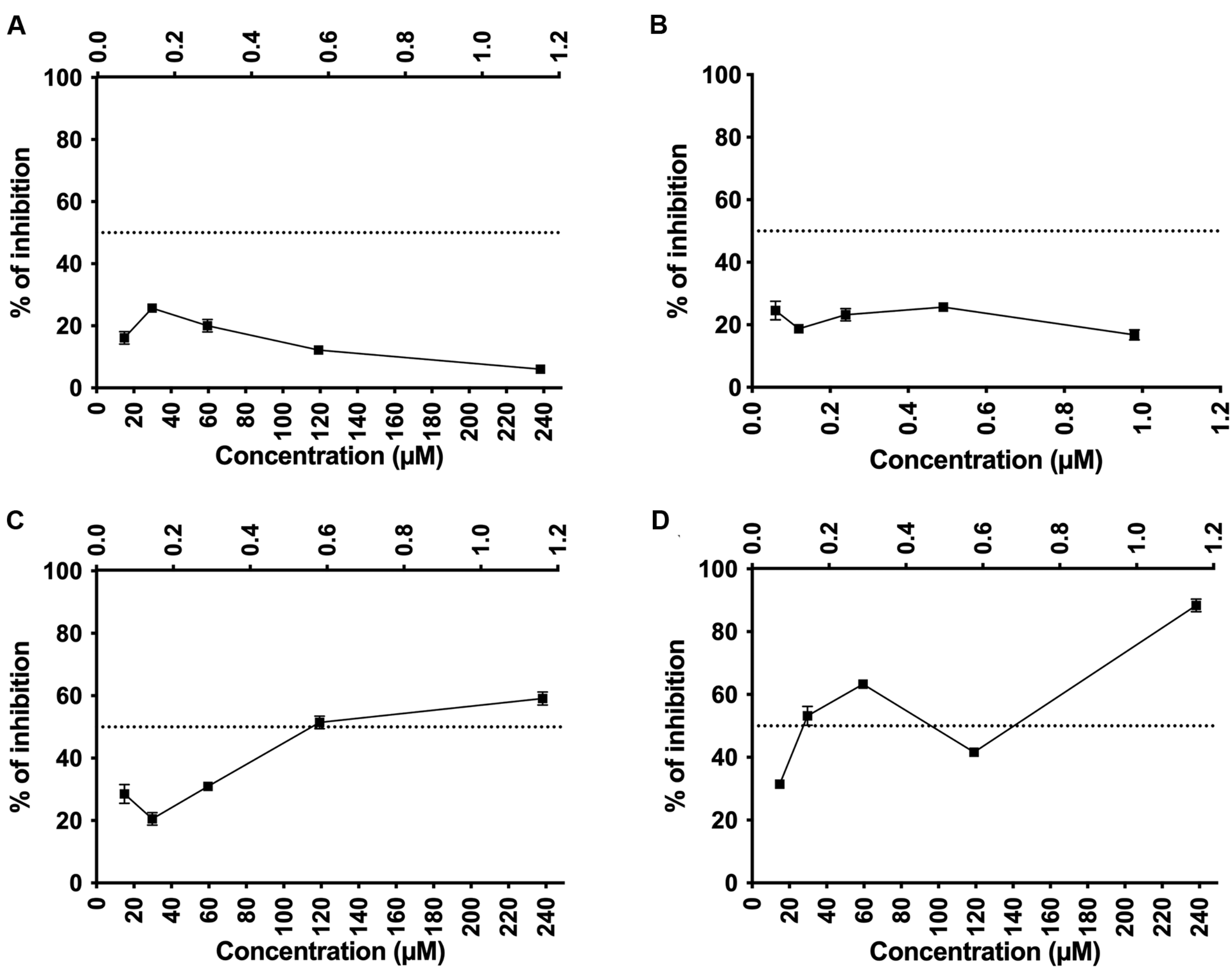

Fig. 4: dose-response curves for the determination of IC_{50} values of nanoemulsion formulations against HSV-1 (KOS strain). HSV-1 (KOS strain)-infected VERO cells were treated with increasing concentrations of NE Blank (A), NE RSV (B), NE BCP (C), or NE RSV+BCP (D), and viral inhibition was determined by plaque reduction assay. Data are expressed as mean ± SD of independent experiments. Nonlinear regression analysis was performed to evaluate the concentration-response profiles of the formulations.

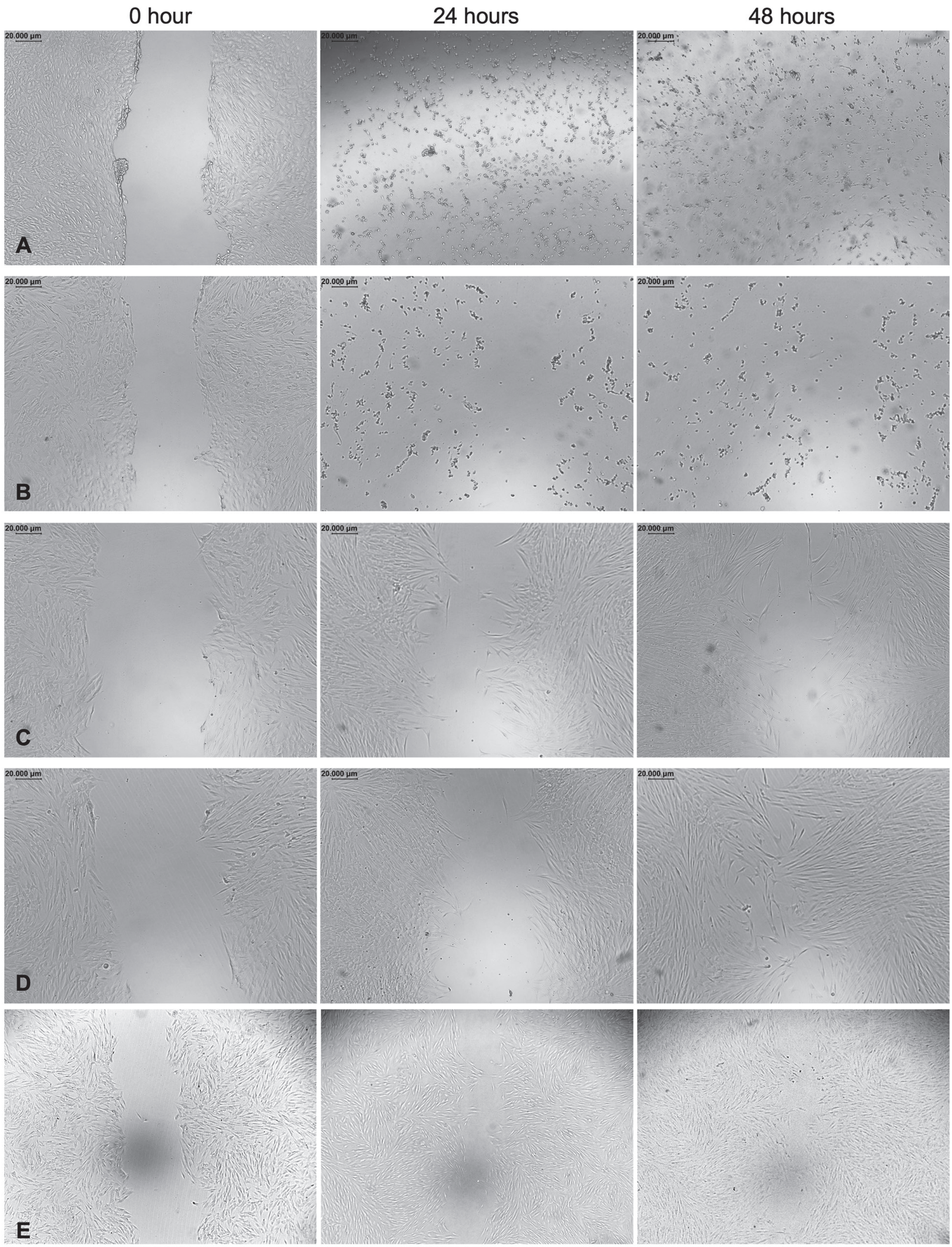

Fig. 5: representative images of the scratch wound-healing assay in HGF cells at 0, 24, and 48 hours after treatment. (A) NE RSV+BCP, (B) NE BCP, (C) NE RSV, (D) untreated cell control, and (E) blank nanoemulsion (NE Blank). Images were obtained immediately after scratch formation (0 h) and after 24 and 48 h of incubation to assess cell migration and wound closure.
