# Supplementary material for: Nanoemulsions loaded with ß-caryophyllene and resveratrol for herpes infections
Source: Mem Inst Oswaldo Cruz. 2026 Oct 12;121:e250333. doi: 10.1590/0074-02760250333 (PMC13660975; doi:10.1590/0074-02760250333)
Supplement: Peer review history

# Reviewer report: FIRST REVIEW ROUND - REVIEWERS’ COMMENTS

## REVIEWER #1

The article “NANOEMULSIONS LOADED WITH β-CARYOPHYLLENE AND RESVERATROL FOR HERPES INFECTIONS” presents a valuable scientific contribution. The study addresses a relevant topic by evaluating nanoemulsions loaded with β-caryophyllene and resveratrol as an alternative approach for the treatment of herpes infections.

a) Adequacy of the abstract: The abstract is well structured, clear, and effectively summarizes the objectives, methodology, main results, and conclusions of the study.

b) Originality and importance of the contribution:

The work demonstrates originality and represents an important contribution to the advancement of the field, particularly in the development of nanoemulsion-based systems for bioactive compounds with antiviral potential.

c) Methodology, results, and discussion:

The methodology is appropriate and well described, and the results are relevant and consistent with the objectives of the study. The discussion adequately interprets the findings and places them within the context of existing literature.

It is suggested that the following comments be taken into consideration:

Page 6, lines 37 and 41: replace “Singh and collaborators” with “Sing et al.” and “Santos Porto and collaborators” with “Santos Porto et al.”

Page 9, line 55: replace “Suarez-Arnedo and collaborators” with “Suarez-Arnedo et al.” The same suggestion applies to page 15, line 39.

Page 6, lines 54–56: According to Table I, 25 mL of ethanol and 50 mL of Milli-Q water are used. The wording of “Finally, ethanol was removed under reduced pressure at 32 °C, resulting in a final volume of formulation of 10 mL” should be improved, as it is not clear how the water would evaporate at 32 °C.

Tables III, IV, and V: The number of replicates and the standard deviation should be reported.

d) References: The references are relevant, up to date, and appropriately support the background and discussion of the study. It is suggested that the following comments be taken into consideration: The introduction may benefit from the inclusion of a paragraph discussing nanoemulsions and their use for encapsulating bioactive compounds, such as β-caryophyllene and resveratrol.

e) Figures and tables: The figures and tables are generally clear and informative. However, in Tables III, IV, and V, the number of replicates and the corresponding standard deviation should be reported to improve the clarity and statistical robustness of the results.

## REVIEWER #2

The manuscript by Dai Prá et al. entitled “NANOEMULSIONS LOADED WITH β-CARYOPHYLLENE AND RESVERATROL FOR HERPES INFECTIONS” is a study that evaluate potential therapies for Herpes.

Even if the contents can have some interest for the reader, in my opinion, several issues have to be solved before considering the manuscript suitable for publication.

The paper is generally unclear. A more organized presentation can improve the message significantly, clearly stating the objectives, methods, and results. Special attention should be given to the results and discussion, since there are a lot of missing results and the ones that are present in the paper are not fully compared to the literature. Also, the efficacy of the test treatment is unclear, and more experiments should be done to evaluate it.

Abstract

The conclusion to the abstract is ambiguous for the results presented in the paper.

Keyword:

All the keywords are in the abstract or tite. I suggest choosing a different set of words tconducto provide more research and locate the published work.

Introduction

Pg 5, line 39: Besides WHO recognizing the need to develop new therapies against HSV, the paper does not show the relevance in it, since they cite the long-term prophylactic treatment, but how common is this kind of treatment? How many HSV strains are drug-resistant? A clearer explanation of these parts is important to justify the work’s relevance.

Pg 5, line 41: the citation of the sentence isn’t of the WHO or Herpes Cure Advocacy. It is relevant to cite them directly in this case.

Pg 5, line 45: “…as they are usually safer or result in lower toxicity than synthetic ones.” This affirmation is controversial, since lots of commercial and illegal drugs are derived from plant-based compounds. Therefore, at least, a citation to support this affirmation is important.

Pg 6, line 29: It isn’t clear in the introduction why nanotechnology is necessary to vector the RSV and BCP, since these make the process more expensive.

Pg 6, line 32: There is a lot of work with these molecules nanoencapsulated, and they are not cited, nor is the nanoemulsion used for this purpose. In the introduction, the only part that mentions the nanoemulsion provides no references to support it.

Methodology:

Pg 6, line 46: I suggest that the specific medium-chain triglycerides be specified.

Pg 7, line 54: the final volume is lower than the initial volume of water, so ethanol and water must be removed.

Pg 8, line 16: since you dilute the sample to analyse in the DLS, how do you guarantee that the increase in water does not impact the final volume of the sample? I suggest you characterize the sample using a methodology that does not require such dilution, such as Cryo-TEM or SAXS.

Pg 11, line 16: “All assays were performed in triplicate The 50% cytotoxic concentration (CC50)”. It is missing a dot between the words “triplicate” and “The”.

An experiment to evaluate how much RSV and BCP are encapsulated and the quality of these components is important to ensure that the results are due to the drug and not to degradation products that may occur during the process.

Results:

Pg 12, line 35: After the sentence, write (data not shown).

Pg. 12, line 37: In the first paragraph of item 3.3, the results are described, but no figure or table presenting them is shown. Therefore, I suggest including a figure with graphs in the paper to better illustrate the results, especially the comparison between the nanoformulation and the free drug. This is important because some conclusions are unsupported by the paper, and it is unclear how the doses used in the antiviral test were selected.

Pg 13: Figures 2 and 3 could have clearer legends with more details on how the statistics are calculated and whether the graphs show standard deviation or standard error. Also, the resolution of the figures is low.

Pg13: The chosen doses for the antiviral activity are not well explained, since they are much lower than the IC50 in the VERO cells.

Pg 13, line 38: The first paragraph of the item 3.4 Wound-healing assay and UV protection capacity” are discussion and not results.

Pg. 14, line 16: I suggest including the CC50 value for VERO in Table III to make comparisons easier. In the table, it is important to include the µM symbol, not only in the legend.

The cytotoxicity results in HGF are not presented. These data are essential because they guide the dose selection for subsequent experiments.

Pg 14, line 28: The data are not shown in figures or tables. Therefore, it is not possible to evaluate. Also, it would be nice to have optical microscopy images for the scratch assay.

Pg 14, line 44: The results shown in Figure 5 are contrary to the sentence.

Pg 15, line 26: In the title of Table IV, describe the dose used for the material in the test.

Since there was a detachment of the cell treated with NE BCP and NE BCP+RSV but not in the NE RSV, it would be important to repeat this experiment using blank NE and Free drug BCP to observe if this happens because of the BCP dose or because of the NE.

Pg15, line 18: The results for the NE RSV+BCP are not shown for these tests. Why did the authors choose not to test them?

Discuss:

Pg 16, lines 9: The value of the Zeta potential of the NE Blank are type different from the one described in Table II.

Pg 16, line 14: It is important to cite supporting literature for the group hypothesis. Therefore, I suggest the addition of a literature reference in the first paragraph of the discussion. Here are some suggestions: Hemlata et al. (2025) – doi: 10.1093/biomethods/bpaf066.; Muntimadugu et al. (2022) – doi: 10.3390/ijms23074034; Guo et al. (2021) – doi: 10.3390/nano11051293.

Pg 16, line 39: The dose of RSV reported in the literature cited in this work is higher than the dose used for the cell viability study. Therefore, it is unclear why the dose range of 0.03-15.63 µM was chosen for the study of cell viability.

Pg. 17, line 24: Since this work did not study the mechanism of action of the drugs BCP and RSV on the viruses, it is important that the appropriate literature be cited in this paragraph.

Pg 18, line 6: Again, the appropriate literature should be cited.

Pg 18, line 12: No statistical difference was observed in the NE RSV when compared to the control group in 24h at the wound healing studies. Therefore, this slight increase is insignificant.

Pg 18, line 42: Since the work of KK, Aathira et al (2024) is in silico and does not evaluate the nanoparticle of BCP, only free BCP, the comparison with the group work should be done carefully.

Pg 20, line 39. “…contrasts with studies demonstrating significant biological activities of this compound..” cite the studies.

In the discussion section, an explanation should be added on why different methodologies of evaluation of citizenship were used. Because the methodologies for HGF and VERO differ, it is not possible to compare the results.

It should also include an explanation of why the selected RSV doses for wound closure were 10, 20, and 40 µm. Also, the results for these experiments

## EDITOR COMMENTS:

1. The authors must substantively address the main points raised by Reviewer 2 regarding the methodology and experimental design.

2. In Table III, it is necessary to correct the CC50 value (1.37 + 335.2) of the “NE RSV+BCP” condition. Furthermore, as requested by Reviewers 1 and 2, all tables must contain the mean and standard deviation, as well as the number of experiments performed.

3. “Figure 2. Effect of the RSV, BCP and RSV+BCP on HSV-1 (KOS strain) in VERO cells (n=3) deviation”. As requested by Reviewer 2, this figure must contain the mean and standard deviation.

4. “and the RSV+BCP combination was tested at the same concentrations in a fixed ratio (1:244).” Why was the combination used at that specific concentration? This should be clear to the readers.

5. The authors use “min” and “minutes”, for example in “for 30 min” and “ 50 mJ/cm² for 30 minutes”. The authors should standardize this throughout the manuscript. The same applies to “hours” and “h”. Standardization is necessary.

6. The abbreviation and company name for fetal bovine serum should be used first in “and supplemented with 10% fetal bovine serum (Gibco).” Afterwards, use only the abbreviation.

7. The citation format throughout the manuscript needs to be modified. Please remove the year of publication from all ‘et al.’ citations, particularly in the Materials and Methods and Discussion sections, to adhere to the journal’s formatting guidelines.

## AUTHORS’ RESPONSE TO THE REVIEWERS

The Editorial Office

Memórias do Instituto Oswaldo Cruz

Dear Editor,

Thank you very much for your interest in our manuscript “Nanoemulsions loaded with β-caryophyllene and resveratrol for herpes infections”.

We are very grateful for the comments and suggestions of the reviewers and we have revised our manuscript accordingly. The formatting alterations and modifications in the text were performed and saved in the file “editor corrections”.

The improvements and comments are described below in a detailed point-by-point response.

We thank you and the reviewers for the critical and valuable comments. We believe that they have helped us to improve the manuscript substantially and we hope that the manuscript is now acceptable for publication in *Memórias do Instituto Oswaldo Cruz*.

Sincerely yours,

Professor Izabella Thaís Silva, PhD

## REVIEWER COMMENTS:

Reviewer 1:

The article “NANOEMULSIONS LOADED WITH β-CARYOPHYLLENE AND RESVERATROL

FOR HERPES INFECTIONS” presents a valuable scientific contribution. The study addresses a relevant topic by evaluating nanoemulsions loaded with β-caryophyllene and resveratrol as an alternative approach for the treatment of herpes infections.

Adequacy of the abstract: The abstract is well structured, clear, and effectively summarizes the objectives, methodology, main results, and conclusions of the study.

Originality and importance of the contribution: The work demonstrates originality and represents an important contribution to the advancement of the field, particularly in the development of nanoemulsion-based systems for bioactive compounds with antiviral potential.

Methodology, results, and discussion: The methodology is appropriate and well described, and the results are relevant and consistent with the objectives of the study. The discussion adequately interprets the findings and places them within the context of existing literature.

It is suggested that the following comments be taken into consideration:

Page 6, lines 37 and 41: replace “Singh and collaborators” with “Sing et al.” and “Santos Porto and collaborators” with “Santos Porto et al.”

Response: Thank you for this suggestion. The requested adjustments have been made in the revised manuscript.

Page 9, line 55: replace “Suarez-Arnedo and collaborators” with “Suarez-Arnedo et al.” The same suggestion applies to page 15, line 39.

Response: Thank you for this suggestion. The requested adjustments have been made in the revised manuscript.

Page 6, lines 54–56: According to Table I, 25 mL of ethanol and 50 mL of Milli-Q water are used. The wording of “Finally, ethanol was removed under reduced pressure at 32 **°**C, resulting in a final volume of formulation of 10 mL” should be improved, as it is not clear how the water would evaporate at 32 °C.

Response: Thank you for pointing this out. We agree that the original wording was unclear. The reduced pressure applied during rotary evaporation was sufficient to remove not only the ethanol (organic phase) but also part of the water, thereby concentrating the formulation to a final volume of 10 mL. To avoid ambiguity, we have revised the sentence as follows:

“Finally, the organic solvent and excess water were removed under reduced pressure at 215 rpm and 32 °C, resulting in a final formulation volume of 10 mL.”

Tables III, IV, and V: The number of replicates and the standard deviation should be reported.

Response: Thank you for this suggestion. The standard deviation (SD) values have been added to Tables III, IV, V, and VI to better represent data variability and improve the clarity of the cytotoxicity results.

References: The references are relevant, up to date, and appropriately support the background and discussion of the study. It is suggested that the following comments be taken into consideration: The introduction may benefit from the inclusion of a paragraph

discussing nanoemulsions and their use for encapsulating bioactive compounds, such as **β**-caryophyllene and resveratrol.

Response: Thank you for this suggestion. We agree that the introduction would benefit from a dedicated paragraph contextualizing nanoemulsions and their relevance for encapsulating BCP and RSV. The following paragraph has been added at the end of the Introduction, immediately before the final paragraph stating the study objectives:

*“Nanoemulsions are colloidal dispersions composed of two immiscible phases, typically oil and wate, stabilized by surfactants and co-surfactants, with droplet sizes generally ranging from 10 to 200 nm. Their nanometric size confers unique properties, including a large interfacial surface area and enhanced kinetic stability, which contribute to improved solubility, bioavailability, and controlled release of encapsulated bioactive compounds (Barradas & Holanda e Silva, 2021; Li et al., 2023). These characteristics make nanoemulsions particularly suitable for the delivery of lipophilic natural compounds.*

*In this context, nanoencapsulation represents a promising strategy for compounds such as* β*-caryophyllene (BCP) and resveratrol (RSV), which present important pharmaceutical limitations. BCP is highly susceptible to air oxidation, leading to the formation of caryophyllene oxide, a less biologically active derivative, while its volatility further reduces therapeutic efficacy during storage (Sköld et al., 2006; Santos Porto et al., 2024). RSV, in turn, suffers from poor aqueous solubility, photoisomerization from the active trans- to the inactive cis-form, and extensive first-pass metabolism, which severely limit its bioavailability (Li et al., 2023; Devi et al., 2019). Nanoemulsions prepared by low-energy methods, such as spontaneous emulsification, provide a protective lipid environment that minimizes degradation while enhancing interaction with biological membranes and facilitating cellular uptake (Barradas & Holanda e Silva, 2021; Pavoni et al., 2020). Therefore, nanoemulsions emerge as a rational strategy to improve the therapeutic performance of RSV and BCP.*

*Considering the variety of pharmacological activities described above and the already known safety profile of these compounds, the hypothesis of this study was to investigate whether both could act synergistically against HSV. Additionally, this study aimed to evaluate whether nanoencapsulation of these active compounds could improve their anti-herpetic activity.”*

Figures and tables: The figures and tables are generally clear and informative. However, in Tables III, IV, and V, the number of replicates and the corresponding standard deviation should be reported to improve the clarity and statistical robustness of the results.

Response: Thank you for this suggestion. Standard deviations and a note indicating that the values represent the mean of three independent experiments (n = 3) have been added to Tables III, IV, and V in the revised manuscript.

## Reviewer 2:

The manuscript by Dai Prá et al. entitled “NANOEMULSIONS LOADED WITH β-CARYOPHYLLENE AND RESVERATROL FOR HERPES INFECTIONS” is a study that evaluate potential therapies for Herpes. Even if the contents can have some interest for the reader, in my opinion, several issues have to be solved before considering the manuscript suitable for publication.

The paper is generally unclear. A more organized presentation can improve the message significantly, clearly stating the objectives, methods, and results. Special attention should be given to the results and discussion, since there are a lot of missing results and the ones that are present in the paper are not fully compared to the literature. Also, the efficacy of the test treatment is unclear, and more experiments should be done to evaluate it.

Abstract

The conclusion to the abstract is ambiguous for the results presented in the paper.

Response: Thank you for this comment. We agree that the conclusion of the abstract could be clearer and more directly reflect the results of the study. Accordingly, the final sentence of the abstract has been revised to explicitly highlight the enhanced antiviral activity observed for the combined RSV and BCP nanoemulsions, indicating a synergistic effect between both compounds. The revised version also clarifies the effects of nanoencapsulation on cytotoxicity and acknowledges the lack of significant impact on wound healing and UV protection assays. These changes have been incorporated in the revised manuscript.

Keyword: All the keywords are in the abstract or title. I suggest choosing a different set of words tconducto provide more research and locate the published work.

Response: Thank you for this suggestion. The requested adjustments have been made in the revised manuscript.

Introduction

Pg 5, line 39: Besides WHO recognizing the need to develop new therapies against HSV, the paper does not show the relevance in it, since they cite the long-term prophylactic treatment, but how common is this kind of treatment? How many HSV strains are drug-resistant? A clearer explanation of these parts is important to justify the work’s relevance.

Pg 5, line 41: the citation of the sentence isn’t of the WHO or Herpes Cure Advocacy. It is relevant to cite them directly in this case.

Pg 5, line 45: “…as they are usually safer or result in lower toxicity than synthetic ones.” This affirmation is controversial, since lots of commercial and illegal drugs are derived from plant-based compounds. Therefore, at least, a citation to support this affirmation is important.

Pg 6, line 29: It isn’t clear in the introduction why nanotechnology is necessary to vector the RSV and BCP, since these make the process more expensive. Pg 6, line 32: There is a lot of work with these molecules nanoencapsulated, and they are not cited, nor is the nanoemulsion used for this purpose. In the introduction, the only part that mentions the nanoemulsion provides no supporting references.

Response: Thank you for these comments. The Introduction has been revised to improve clarity and strengthen the justification of the study. Additional information on long-term suppressive therapy and the prevalence of drug-resistant HSV strains has been included. The statement regarding the need for new therapies has been corrected to directly cite the World Health Organization. The description of plant-derived compounds has been revised to avoid overgeneralization and is now supported by an appropriate reference. Furthermore, the rationale for nanoencapsulation has been clarified based on the physicochemical limitations of RSV and BCP, and a dedicated paragraph on nanoemulsions, supported by relevant references, has been added. These changes have been incorporated in the revised manuscript.

Methodology:

Pg 6, line 46: I suggest that the specific medium-chain triglycerides be specified.

Response: Thank you for this comment. The specific medium-chain triglyceride used in this study has been specified in the Materials section.

Pg 7, line 54: the final volume is lower than the initial volume of water, so ethanol and water must be removed.

Response: Thank you for this observation. The methodology has been revised to clarify that both ethanol and excess water were removed under reduced pressure to obtain the final formulation volume. This information has been included in the revised manuscript.

Pg 8, line 16: since you dilute the sample to analyse in the DLS, how do you guarantee that the increase in water does not impact the final volume of the sample? I suggest you characterize the sample using a methodology that does not require such dilution, such as Cryo-TEM or SAXS.

Response: Thank you for this important comment. Although dilution is required for DLS measurements, this is a standard and widely accepted procedure to minimize multiple scattering effects without significantly altering droplet size when appropriate dilution factors are used [Bhattacharjee, 2016; McClements, 2012]. In this study, a low dilution ratio (1:10, v/v) was applied, which is commonly reported for nanoemulsion systems. Moreover, DLS is a well-established technique for determining hydrodynamic diameter and polydispersity index, providing reliable information on size distribution and colloidal stability [Kumar, 2017]. Considering the scope of this study, DLS was considered an appropriate method for physicochemical characterization.

Pg 11, line 16: “All assays were performed in triplicate The 50% cytotoxic concentration (CC50)”. It is missing a dot between the words “triplicate” and “The”.

Response: Thank you for pointing this out. The sentence has been corrected in the revised manuscript.

An experiment to evaluate how much RSV and BCP are encapsulated and the quality of these components is important to ensure that the results are due to the drug and not to degradation products that may occur during the process.

Response: Thank you for this important comment. We agree that the evaluation of encapsulation efficiency is relevant for a comprehensive characterization. However, the primary objective of this study was to assess the biological activity of the nanoemulsions, particularly their antiviral and cytotoxic effects.

Given the lipophilic nature of RSV and BCP, their incorporation into the oil phase of nanoemulsions is expected and has been widely reported in the literature, with high incorporation efficiency typically observed for similar compounds [1–3]. Additionally, nanoemulsion systems have been extensively described as suitable carriers for improving the stability and delivery of lipophilic bioactives such as resveratrol [4].

Furthermore, the consistent physicochemical properties observed (particle size, PDI, and zeta potential), together with the biological results obtained, support the effective incorporation of the compounds in the developed systems.

Donsì F, Sessa M, Mediouni H, Mgaidi A, Ferrari G. Encapsulation of bioactive compounds in nanoemulsion- based delivery systems. Procedia Food Sci. 2011;1. https://doi.org/10.1016/j.profoo.2011.09.246

Gupta A, Eral HB, Hatton TA, Doyle PS. Controlling and predicting droplet size of nanoemulsions: Scaling relations with experimental validation. Soft Matter. 2016;12. https://doi.org/10.1039/c5sm02051d

Santos Porto D, da Costa Bernardo Port B, Conte J, Fretes Argenta D, Pereira Balleste M, Amadeu Micke G, et al. Development of ophthalmic nanoemulsions of β-caryophyllene for the treatment of Acanthamoeba keratitis. Int J Pharm. Elsevier B.V.; 2024;659. https://doi.org/10.1016/j.ijpharm.2024.124252

R. Neves A, Lucio M, L.C. Lima J, Reis S. Resveratrol in Medicinal Chemistry: A Critical Review of its Pharmacokinetics, Drug-Delivery, and Membrane Interactions. Curr Med Chem. 2012;19. https://doi.org/10.2174/092986712799945085

Results:

Pg 12, line 35: After the sentence, write (data not shown).

Response: Thank you for this suggestion. It has been added in the revised manuscript.

Pg. 12, line 37: In the first paragraph of item 3.3, the results are described, but no figure or table presenting them is shown. Therefore, I suggest including a figure with graphs in the paper to better illustrate the results, especially the comparison between the nanoformulation and the free drug. This is important because some conclusions are unsupported by the paper, and it is unclear how the doses used in the antiviral test were selected.

Response: We thank the reviewer for this suggestion. A new table (Table III) has been created presenting the CC_y0_ values together with their respective 95% confidence intervals (CI_9y_%), allowing a clear and direct comparison between the nanoformulations and the free compounds. Based on the CC_y0_ values obtained, a preliminary antiviral assay was performed using concentrations below the cytotoxic threshold. Subsequently, the concentrations used in the antiviral assays presented in the manuscript were defined according to the results of this preliminary screening.

Pg 13: Figures 2 and 3 could have clearer legends with more details on how the statistics are calculated and whether the graphs show standard deviation or standard error. Also, the resolution of the figures is low.

Response: Thank you for this comment. The legends of figures 2 and 3 have been revised to include more detailed descriptions of the experimental model, including the cell line, viral assay (plaque reduction assay), and data interpretation. The legends now clearly indicate how the data are expressed and include the number of replicates and variability (mean ± standard deviation). As these figures represent dose–response antiviral activity profiles, no inferential statistical analysis was applied. In addition, the figures have been improved in terms of resolution and readability in the revised manuscript.

Pg13: The chosen doses for the antiviral activity are not well explained, since they are much lower than the IC50 in the VERO cells.

Response: Thank you for this comment. The concentrations used in the antiviral assays were selected based on the CC_y0_ values in VERO cells, ensuring that only non-cytotoxic concentrations were tested. This approach is essential to distinguish true antiviral effects from cytotoxicity. The IC_y0_ values were determined from the concentration–response curves obtained within this non-cytotoxic range.

Pg 13, line 38: The first paragraph of the item 3.4 Wound-healing assay and UV protection capacity” are discussion and not results.

Response: The reviewer is correct in this observation. The first paragraph of section 3.4 included interpretative aspects that are more suitable for the discussion section. Accordingly, this paragraph has been removed from the results section and the corresponding considerations have been incorporated into the discussion.

Pg. 14, line 16: I suggest including the CC50 value for VERO in Table III to make comparisons easier. In the table, it is important to include the µM symbol, not only in the legend.

The cytotoxicity results in HGF are not presented. These data are essential because they guide the dose selection for subsequent experiments.

Response: We thank the reviewer for this suggestion. The µM unit has now been added directly to the cytotoxicity values presented in the tables to improve clarity. In addition, a new table (Table III) has been created to present the CC_y0_ values for VERO cells, allowing a clearer comparison between free compounds and nanoformulations. The cytotoxicity results in HGF cells are now fully presented in Table IV, and the standard deviation (SD) values have been included in Tables III, IV, V, and VI to better represent data variability.

Pg 14, line 28: The data are not shown in figures or tables. Therefore, it is not possible to evaluate. Also, it would be nice to have optical microscopy images for the scratch assay.

Response: We thank the reviewer for this important comment. We would like to clarify that the observations described in this section correspond to preliminary experiments performed using concentrations selected based on the antiviral activity results (0.98 µM NE RSV and 238.00 µM NE BCP). In these initial conditions, treatment with NE BCP and NE RSV+BCP resulted in a pronounced loss of cell adhesion, which prevented quantitative analysis of wound closure. Therefore, no numerical data are presented for these groups, and the results are described qualitatively as an indication of impaired cell adhesion and migration. In contrast, this effect was not observed for NE RSV, for which cell adhesion was preserved, allowing quantitative evaluation of wound closure, and the corresponding values are presented in the text.

Pg 14, line 44: The results shown in Figure 5 are contrary to the sentence.

Response: We thank the reviewer for this careful observation. We acknowledge that, in figure 5, the highest concentration of NE RSV (40 µM) showed a statistically significant difference compared to the untreated control. However, this difference corresponds to a lower wound closure percentage relative to the control, indicating no enhancement of the healing process. Since this experiment aimed to evaluate the potential of the nanoemulsions to promote wound healing, this effect was not interpreted as a positive outcome.

Importantly, no statistically significant differences were observed between the different concentrations tested for either NE RSV or NE BCP. Therefore, the conclusion that the nanoemulsions did not promote wound healing within the tested concentration range remains valid.

Pg 15, line 26: In the title of Table IV, describe the dose used for the material in the test.

Since there was a detachment of the cell treated with NE BCP and NE BCP+RSV but not in the NE RSV, it would be important to repeat this experiment using blank NE and Free drug BCP to observe if this happens because of the BCP dose or because of the NE.

Response: We thank the reviewer for this valuable suggestion. The title of Table V has been revised to explicitly include the concentrations of the tested formulations, as requested.

In addition, we would like to clarify that the blank nanoemulsion (NE blank) was evaluated under the same experimental conditions, and cell viability values remained comparable to those of the untreated control group, indicating that the nanoemulsion system itself did not induce loss of cell viability or adhesion.

Therefore, the pronounced loss of cell adhesion observed for NE BCP and NE RSV+BCP is likely associated with the presence and concentration of β-caryophyllene rather than the nanoemulsion system itself.

Pg15, line 18: The results for the NE RSV+BCP are not shown for these tests. Why did the authors choose not to test them?

Response: We thank the reviewer for this comment. NE RSV+BCP was initially evaluated under UV exposure conditions; however, no improvement in cell viability was observed under these conditions.

Based on these observations, additional assays were designed to investigate whether different concentration ranges of the individual nanoemulsions (NE RSV and NE BCP) could improve cell viability under UV exposure. However, no beneficial effects were observed for either formulation.

Therefore, the combined formulation (NE RSV+BCP) was not further evaluated in these subsequent assays, as the individual components did not demonstrate improved performance.

Discuss:

Pg 16, lines 9: The value of the Zeta potential of the NE Blank are type different from the one described in Table II.

Response: We thank the reviewer for carefully identifying this inconsistency. The difference between the zeta potential value reported in the Discussion section and that presented in Table II resulted from an inconsistency in the text. The correct value for the zeta potential of the NE Blank is −19.70 ± 3.37 mV, as shown in Table II.

Pg 16, line 14: It is important to cite supporting literature for the group hypothesis. Therefore, I suggest the addition of a literature reference in the first paragraph of the discussion. Here are some suggestions: Hemlata et al. (2025) – doi: 10.1093/biomethods/bpaf066.; Muntimadugu et al. (2022) – doi: 10.3390/ijms23074034; Guo et al. (2021) – doi: 10.3390/nano11051293.

Response: We thank the reviewer for this suggestion. Supporting references have now been added to the first paragraph of the Discussion to substantiate the proposed hypothesis, without altering the original interpretation of the results. These references support the relationship between particle size distribution and colloidal stability, as well as the role of surface charge and interfacial interactions in lipid-based nanosystems.

Pg 16, line 39: The dose of RSV reported in the literature cited in this work is higher than the dose used for the cell viability study. Therefore, it is unclear why the dose range of 0.03-15.63 µM was chosen for the study of cell viability.

Response: We thank the reviewer for this important observation. The concentration range used for RSV (0.03–15.63 µM) was selected based on preliminary experiments aimed at identifying non-cytotoxic concentrations suitable for subsequent antiviral assays. Since the main objective of this study was to evaluate antiviral activity under biologically relevant and non-toxic conditions, the selected range ensured cell viability above 75%, avoiding confounding effects related to cytotoxicity. Additionally, this concentration range was defined considering its use in the RSV+BCP combination, in which the compounds were evaluated at a fixed ratio, ensuring that both could be tested simultaneously within a non-cytotoxic and comparable concentration window.

Pg. 17, line 24: Since this work did not study the mechanism of action of the drugs BCP and RSV on the viruses, it is important that the appropriate literature be cited in this paragraph.

Response: We thank the reviewer for this comment. Appropriate literature describing the antiviral mechanisms of RSV and BCP has been added to this paragraph.

Pg 18, line 6: Again, the appropriate literature should be cited.

Response: We thank the reviewer for this comment. Appropriate literature supporting the interpretation of the combinatorial effect has been added to this sentence.

Pg 18, line 12: No statistical difference was observed in the NE RSV when compared to the control group in 24h at the wound healing studies. Therefore, this slight increase is insignificant.

Response: We thank the reviewer for this comment. The manuscript has been revised to clarify that no statistically significant difference was observed for NE RSV compared to the control group at 24 h, and the interpretation of a potential effect has been removed.

Pg 18, line 42: Since the work of KK, Aathira et al (2024) is in silico and does not evaluate the nanoparticle of BCP, only free BCP, the comparison with the group work should be done carefully.

Response: We thank the reviewer for this comment. We agree that the study by KK, Aathira et al. (2024) evaluates the effects of free β-caryophyllene, whereas our work investigates nanoencapsulated BCP. Therefore, the comparison should be interpreted with caution.

The manuscript has been revised to clarify this distinction and to avoid direct comparison between free and nanoencapsulated forms, emphasizing the differences in formulation and their potential impact on biological responses.

Pg 20, line 39. “…contrasts with studies demonstrating significant biological activities of this compound..” cite the studies.

Response: We thank the reviewer for this comment. Appropriate references supporting the biological activity of β-caryophyllene have been added to this sentence.

In the discussion section, an explanation should be added on why different methodologies of evaluation of citizenship were used. Because the methodologies for HGF and VERO differ, it is not possible to compare the results.

Response: We thank the reviewer for this important comment. The use of different cytotoxicity assays was based on the specific characteristics of each cell line. The sulforhodamine B (SRB) assay was used for VERO cells, while the MTT assay was employed for HGF cells, as these methodologies are well established and routinely used for these respective cell lines in our research group.

Each assay was applied consistently within its respective experimental model, and the results were interpreted independently. No direct comparison between VERO and HGF data was intended in the manuscript.

It should also include an explanation of why the selected RSV doses for wound closure were 10, 20, and 40 µm. Also, the results for these experiments

Response: We thank the reviewer for this comment. The concentrations of NE RSV (10, 20, and 40 µM) used in the wound-healing assay were selected following the same rationale applied in the UV protection experiments. Since no significant effect was observed at the antiviral concentration, higher concentrations of RSV were tested to investigate whether increased doses could promote wound healing activity.

## EDITOR COMMENTS:

The authors must substantively address the main points raised by Reviewer 2 regarding the methodology and experimental design.

Response: We thank the editor for the valuable comments. The points raised regarding the methodology and experimental design have been carefully addressed and revised throughout the manuscript.

In Table III, it is necessary to correct the CC50 value (1.37 + 335.2) of the “NE RSV+BCP” condition. Furthermore, as requested by Reviewers 1 and 2, all tables must contain the mean and standard deviation, as well as the number of experiments performed.

“Figure 2. Effect of the RSV, BCP and RSV+BCP on HSfV-1 (KOS strain) in VERO cells (n=3) deviation”. As requested by Reviewer 2, this figure must contain the mean and standard deviation.

Response: We thank the editor for this comment. Figure 2 has been revised to include the mean values and their corresponding standard deviations, as requested.

“and the RSV+BCP combination was tested at the same concentrations in a fixed ratio (1:244).” Why was the combination used at that specific concentration? This should be clear to the readers.

Response: We thank the editor for this important comment. The fixed ratio (1:244) between RSV and BCP was defined based on the formulation design used in this study. The concentration of BCP was selected according to Santos Porto et al. (2024), in which a concentration of 5.0% was used. In our formulation, BCP was incorporated at 5% to ensure adequate loading in the nanoemulsion system.

For RSV, a working concentration of 500 µM was selected based on its biological activity range. To maintain these target concentrations, the nanoemulsion was prepared containing 5% BCP and 1000 µM RSV (corresponding to 0.02%), resulting in a fixed ratio of 1:244.

This ratio reflects the proportion of both compounds within the formulation and was maintained throughout the experiments to ensure consistency in the evaluation of their combined effects.

The authors use “min” and “minutes”, for example in “for 30 min” and “ 50 mJ/cm² for 30 minutes”. The authors should standardize this throughout the manuscript. The same applies to “hours” and “h”. Standardization is necessary.

Response: We thank the editor for this comment. The manuscript has been revised to standardize the use of time units throughout the text.

The abbreviation and company name for fetal bovine serum should be used first in “and supplemented with 10% fetal bovine serum (Gibco).” Afterwards, use only the abbreviation.

Response: We thank the reviewer for this comment. The manuscript has been revised to introduce fetal bovine serum with its full name and abbreviation at first mention, and to use only the abbreviation (FBS) thereafter.

The citation format throughout the manuscript needs to be modified. Please remove the year of publication from all ‘et al.’ citations, particularly in the Materials and Methods and Discussion sections, to adhere to the journal’s formatting guidelines.

Response: We thank the reviewer for this comment. The citation format has been revised accordingly throughout the manuscript.

# Reviewer report: REVIEWERS’ COMMENTS

## REVIEWER #1

The manuscript by Dai Prá et al., entitled “NANOEMULSIONS LOADED WITH β-CARYOPHYLLENE AND RESVERATROL FOR HERPES INFECTIONS,” presents a study evaluating potential therapies for herpes infections.

The authors addressed all of the questions raised; however, some of the points discussed still give rise to concerns.

Introduction

Pg 4, line 59: Although the reference is very good, the cited article (9) does not mention the use of plant-based compounds as anti-herpetic agents, as stated in the sentence.

Pg 5, line 22: I suggest adding a paragraph beginning with “Both natural compounds...” and merging it with the following paragraph, “Their ability to aid...”

Pg 5, line 38: I believe there is a typo in the word “wate.” I think it should be “water.”

Methodology:

Pg 8, line 36: An experiment to evaluate the extent of RSV and BCP encapsulation, as well as the integrity of these components, is important to ensure that the results are attributable to the drugs themselves rather than to degradation products that may arise during the process. The authors’ response to this point is not satisfactory. Although the methodology used for BCP production is the same as that described in the literature by Santos Porto et al., no study has used the same components at the same concentrations for RSV. Therefore, because the materials that compose the oils vary, the efficiency of their interactions with the components may also vary. In addition, the nanoformulation containing both components may affect encapsulation efficiency. For this reason, it is necessary to conduct a study quantifying encapsulation efficiency and drug content, including to ensure that the doses tested in the cytotoxicity studies correspond to the doses actually administered. Therefore, I strongly recommend conducting a quantification experiment to determine the actual doses.

Results:

Pg 13: The rationale for the doses selected for the antiviral activity assays is not well explained, since they are much lower than the IC₅₀ values in VERO cells.

Response: Thank you for this comment. The concentrations used in the antiviral assays were selected based on the CC₅₀ values in VERO cells, ensuring that only non-cytotoxic concentrations were tested. This approach is essential to distinguish true antiviral effects from cytotoxicity. The IC₅₀ values were determined from the concentration–response curves obtained within this non-cytotoxic range.

I suggest that the curves used for the calculation of the IC₅₀ in cells be shown, perhaps in the supplementary material. I also strongly recommend that, for each compound tested, a dose–response curve be obtained in which at least one concentration produces more than 50% inhibition.

Pg 14, line 2: Although the response states that the reduced wound closure observed at the 40 µM dose of NE RSV demonstrates the absence of a healing effect, a statistically significant difference is present. Therefore, as the manuscript is currently written, it cannot be stated that no statistically significant difference was found, because such a difference does exist. Moreover, the observed reduction in wound closure may suggest that, at this concentration, the healing process is delayed and that it is negatively affected. For this reason, I suggest that these data be analyzed in greater detail.

Pg 15, line 26: In the title of Table IV, please specify the dose of the material used in the test. Since cell detachment was observed in the groups treated with NE BCP and NE BCP+RSV, but not in the NE RSV group, it would be important to repeat this experiment using blank NE and free BCP to determine whether this effect is due to the BCP dose or to the nanoemulsion itself.

Response: We thank the reviewer for this valuable suggestion. The title of Table V has been revised to explicitly include the concentrations of the tested formulations, as requested.

In addition, we would like to clarify that the blank nanoemulsion (NE blank) was evaluated under the same experimental conditions, and cell viability values remained comparable to those of the untreated control group, indicating that the nanoemulsion system itself did not induce loss of cell viability or adhesion.

Therefore, the pronounced loss of cell adhesion observed for NE BCP and NE SV+BCP is likely associated with the presence and concentration of β-caryophyllene rather than the nanoemulsion system itself.

I would like to see the results for the blank nanoparticle, as well as the images acquired for all experimental conditions.

Pg 15, line 18: The results for NE RSV+BCP are not shown for these tests. Why did the authors choose not to evaluate this formulation?

Response: We thank the reviewer for this comment. NE RSV+BCP was initially evaluated under UV exposure conditions; however, no improvement in cell viability was observed under these conditions.

Based on these observations, additional assays were designed to investigate whether different concentration ranges of the individual nanoemulsions (NE RSV and NE BCP) could improve cell viability under UV exposure. However, no beneficial effects were observed for either formulation.

Therefore, the combined formulation (NE RSV+BCP) was not further evaluated in these subsequent assays, as the individual components did not demonstrate improved performance.

This justification should be incorporated into the manuscript.

## REVIEWER #2

No comments.

## AUTHORS’ RESPONSE TO THE REVIEWERS

The Editorial Office

Memórias do Instituto Oswaldo Cruz

Dear Editor,

Thank you very much for your interest in our manuscript ID MIOC-2025 0333.R1 “Nanoemulsions loaded with β-caryophyllene and resveratrol for herpes infections”.

We are very grateful for the comments and suggestions of the reviewers and we have revised our manuscript accordingly. The improvements and comments are described below in a detailed point-by-point response.

We thank you and the reviewers for the critical and valuable comments. We believe that they have helped us to improve the manuscript substantially and we hope that the manuscript is now acceptable for publication in *Memórias do Instituto Oswaldo Cruz*.

Sincerely yours,

Professor Izabella Thaís Silva, PhD

# Reviewer report: REVIEWERS’ COMMENTS

## REVIEWER #1

No comments.

## REVIEWER #2

No comments.
